# Analytical Validation of Multiplex Biomarker Assay to Stratify Colorectal Cancer into Molecular Subtypes

**DOI:** 10.1038/s41598-019-43492-0

**Published:** 2019-05-21

**Authors:** Chanthirika Ragulan, Katherine Eason, Elisa Fontana, Gift Nyamundanda, Noelia Tarazona, Yatish Patil, Pawan Poudel, Rita T. Lawlor, Maguy Del Rio, Si-Lin Koo, Wah-Siew Tan, Francesco Sclafani, Ruwaida Begum, Larissa S. Teixeira Mendes, Pierre Martineau, Aldo Scarpa, Andrés Cervantes, Iain Beehuat Tan, David Cunningham, Anguraj Sadanandam

**Affiliations:** 1 Division of Molecular Pathology, The Institute of Cancer Research, London, United Kingdom; 2 Centre for Molecular Pathology, The Royal Marsden NHS Foundation Trust, London, United Kingdom; 3 CIBERONC, Department of Medical Oncology, Biomedical Research Institute INCLIVA, University of Valencia, Valencia, Spain; 4 ARC-Net Centre for Applied Research on Cancer, University and Hospital Trust of Verona, Verona, Italy; 5 Department of Pathology and Diagnostics, University and Hospital Trust of Verona, Verona, Italy; 6 Institut de Recherche en Cancérologie de Montpellier, Institut National de la Santé et de la Recherche Médicale, U896, Université Montpellier, Centre Régional de Lutte contre le Cancer Val d’Aurelle Paul Lamarque, Montpellier, France; 7 National Cancer Centre Singapore, Singapore, Singapore; 8 Singapore General Hospital, Singapore, Singapore; 9 Department of Medicine, The Royal Marsden NHS Foundation Trust, London, United Kingdom; 10 Genome Institute of Singapore, Singapore, Singapore; 11 Duke-NUS Medical School, Singapore, Singapore

**Keywords:** Tumour biomarkers, Data processing, Tumour heterogeneity, Colorectal cancer, Colorectal cancer

## Abstract

Previously, we classified colorectal cancers (CRCs) into five CRCAssigner (CRCA) subtypes with different prognoses and potential treatment responses, later consolidated into four consensus molecular subtypes (CMS). Here we demonstrate the analytical development and validation of a custom NanoString nCounter platform-based biomarker assay (NanoCRCA) to stratify CRCs into subtypes. To reduce costs, we switched from the standard nCounter protocol to a custom modified protocol. The assay included a reduced 38-gene panel that was selected using an in-house machine-learning pipeline. We applied NanoCRCA to 413 samples from 355 CRC patients. From the fresh frozen samples (n = 237), a subset had matched microarray/RNAseq profiles (n = 47) or formalin-fixed paraffin-embedded (FFPE) samples (n = 58). We also analyzed a further 118 FFPE samples. We compared the assay results with the CMS classifier, different platforms (microarrays/RNAseq) and gene-set classifiers (38 and the original 786 genes). The standard and modified protocols showed high correlation (> 0.88) for gene expression. Technical replicates were highly correlated (> 0.96). NanoCRCA classified fresh frozen and FFPE samples into all five CRCA subtypes with consistent classification of selected matched fresh frozen/FFPE samples. We demonstrate high and significant subtype concordance across protocols (100%), gene sets (95%), platforms (87%) and with CMS subtypes (75%) when evaluated across multiple datasets. Overall, our NanoCRCA assay with further validation may facilitate prospective validation of CRC subtypes in clinical trials and beyond.

## Introduction

Colorectal cancer (CRC) is the fourth leading cause of cancer-related deaths worldwide^1^. The median overall survival of metastatic (m)CRC patients with unresectable disease remains around 24 months with standard chemotherapies. Targeted therapies including anti-EGFR antibodies, anti-angiogenic and immunotherapy agents may extend survival up to 30 months in selected patients^2^. However, selecting patients who will benefit from different standard-of-care treatment regimens remains challenging. Additional predictive biomarkers are required to spare patients from unnecessary toxicities, improve outcomes and increase cost-effectiveness of treatments.

In order to classify CRCs into groups, we previously identified five distinctive gene expression subtypes (CRCAssigner, henceforth referred to as CRCA) using a 786-gene signature: goblet-like, enterocyte, stem-like, inflammatory and transit-amplifying (TA)^3^. We demonstrated significantly poorer disease-free survival (DFS) in untreated patients for the stem-like subtype, intermediate DFS for inflammatory and enterocyte, and better DFS for goblet-like and TA^3^. Then, from two different datasets that included drug response information, we observed increased responses within the stem-like subtype to irinotecan, fluorouracil and leucovorin treatment combination (FOLFIRI), and within the TA subtype to the anti-EGFR monoclonal antibody cetuximab^3–5^ using small sample size cohorts. These treatment responses were further validated by other studies^6,7^. However, additional validation is warranted.

Five other groups independently identified between 3 and 6 molecularly distinct CRC subtypes based on expression profiles^8–12^. To address the ambiguity caused by these independent studies reaching differing conclusions (which could lead to issues with interpretability and generalizability in subsequent studies), these and our findings were aggregated by our CRC Subtyping Consortium (CRCSC) into 4 consensus molecular subtypes (CMS): CMS1 (associated with inflammatory subtype); CMS2 (enterocyte and TA); CMS3 (goblet-like); and CMS4 (stem-like), plus a “mixed” subtype representing either the existence of additional subtypes or the presence of multiple subtypes in a single sample^13^. CRCA and CMS subtypes are generally highly similar (discussed below), with the primary exception that the enterocyte and TA subtypes of CRCA were merged into CMS2 of the consensus classification^13^. The current work for assay development and evaluation of the CRCA subtypes started before we published our CMS subtypes, however the clear connection between these two subtyping schemes suggests that our subtyping assay results are representative of real and consistent biological heterogeneity in CRCs.

Recently, it was reported that addition of oxaliplatin to fluorouracil-leucovorin in early-stage disease revealed an advantage in disease-free survival in the enterocyte subtype compared to the other subtypes^14^. The prospective validation of these findings into routine clinical practice remains challenging, mainly due to the lack of a fit-for-purpose assay that can classify patient samples into subtypes within a clinically relevant turnaround time and reasonable costs using formalin-fixed paraffin-embedded (FFPE) samples. Microarray or RNAseq gene expression profiles for subtype classification are expensive, time consuming, require dedicated bioinformatics expertise, and have turnaround times incompatible with prospective clinical applications. Our previously published proof-of-concept assays - immunohistochemistry (IHC) and quantitative reverse transcriptase polymerase chain reaction (qRT-PCR) methods^3^ - may not be easily applicable when higher numbers of genes are required as biomarkers. Hence, we applied nCounter platform (NanoString Technologies) to develop a clinically-relevant biomarker assay for CRCA subtype classification. A summary of the relative costs and turnaround times of these platforms can be found in Supplementary Table S1a.

The nCounter platform has previously been exploited to develop the Food and Drug Administration (FDA)-approved Prosigna® Breast Cancer Prognostic Gene Signature Assay to predict risk of recurrence in patients treated with adjuvant hormonal therapy^15^, as well as assays to predict medulloblastoma^16^ and lymphoma^17^ subtypes. This platform measures gene expression in the form of discrete counts of barcoded mRNAs, and requires no amplification step, eliminating a potential source of bias. In the present study, we evaluated the suitability of this platform for a gene expression-based assay for our CRCA subtypes using a modified protocol to classify CRCs in multiple independent cohorts (fresh frozen and FFPE samples). The results were compared to the CMS subtype classification and other platforms. A summary of the classifiers utilised in this study is given in Supplementary Table S1b.

## Materials and Methods

### Patient cohorts

Six CRC cohorts of primary tumour samples collected prior to treatment were studied, as summarised in Supplementary Table S1c and detailed below; three derived from fresh frozen, two from FFPE samples and one from matched fresh frozen and FFPE. The first included RNA samples from 17 stage IV patients (Montpellier cohort) from a published study^4^. A second cohort of RNA samples (OriGene; n = 17) was purchased from OriGene (Rockville, MD, USA). A third cohort included 145 fresh frozen samples (Singapore FF) and a fourth cohort included 106 FFPE samples (Singapore FFPE) from patients participating in an on-going observational study. The fifth cohort consisted of 12 FFPE CRC samples from a retrospective tissue collection from The Royal Marsden Hospital, UK (RETRO-C cohort). The sixth and final cohort consisted of 58 stage II-III CRC patients (INCLIVA-Valencia cohort) with matched prospectively collected fresh frozen and FFPE tissue.

### Ethical approval and informed consent

The protocol for the Singapore cohorts (both FF and FFPE) of this study was approved by the SingHealth Institutional Review Board: 2013/110/B. The protocol for the INCLIVA-Valencia cohort was approved by the Comité Etico de Investigacion Clinica del Clínico Universitario de Valencia: F-CE-GEva-15. The Montpellier cohort had ethical approval as described in their original publication and OriGene cohort is available from the commercial vendor. The protocol for the RETRO-C cohort was approved by NRES Committee East of England-Cambridge Central: 10/H0308/28. All patients provided informed consent, and all experiments were performed in accordance with relevant guidelines and regulations.

### Gene sets for nCounter assay

The following 50 genes were initially selected for inclusion in the CRCA subtype custom nCounter assay based on our previous report^3^: (a) seven genes that were proposed in said report as subtype biomarkers for qRT-PCR and IHC^3^; (b) among the top 2 to 9 highest scoring genes for each subtype from predictive analysis of microarrays (PAM) centroids^3^; (c) three additional genes that distinguish TA (cetuximab-sensitive and –resistant) sub-subtypes^3^; and (d) those representing the characteristics of certain subtypes such as WNT signalling, epithelial-mesenchymal transition (EMT)/stroma, MET tyrosine kinase signalling, and NF-κB signalling. An additional 10 housekeeping genes were used for normalization. All genes and relevant annotations are listed in Supplementary Table S1d.

### NanoString nCounter gene expression assays - standard and modified protocols

**Figure 1:**
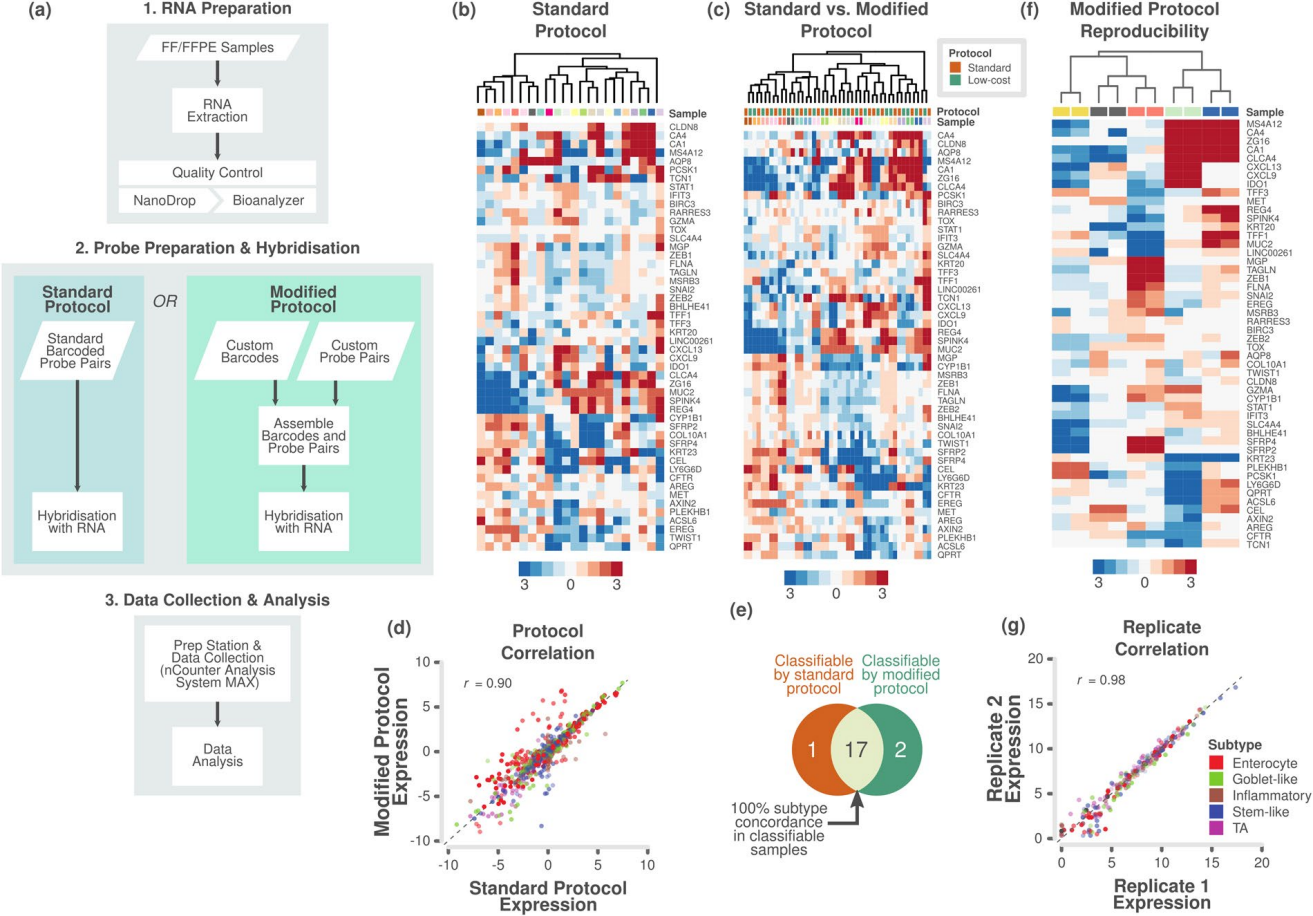
Assessment of different protocols and reproducibility of reduced gene subtype-based nCounter assay in fresh frozen samples. (**a**) Flowchart showing the major steps of the NanoCRCA assay protocols. Specifically, this flowchart demonstrates the difference between standard and modified protocols. Though the modified protocol has additional steps, it substantially reduces the cost without significantly increasing the time of the assay. (**b**,**c**) Heatmap of expression levels of the selected 48 subtype-specific genes (and 2 additional genes) for 22 fresh frozen samples from the Montpellier and OriGene cohorts as measured on a custom nCounter panel using b) standard protocol and c) both standard and modified protocols (median centred within protocols before clustering). (**d**) A scatter plot of gene expression measurements for 48 genes in 22 fresh frozen samples between the standard and modified protocols (median centred within protocols before correlation). Each point is coloured by the gene’s weight and subtype (PAM score) in the CRCA-786 centroids. Correlation co-efficient (Pearson’s *r*) value is shown. (**e**) Venn diagram indicating the number of samples that were classifiable by the standard and modified protocols, and the concordance between classifiable samples. (**f**) Heatmap of expression levels of the selected 48 subtype-specific genes (and 2 additional genes) from technical duplicates of 5 samples assayed using modified protocol with a maximum interval of 40 weeks to show the reproducibility of the assay. (**g**) A scatter plot of gene expression measurements for the 48 genes in 5 technical duplicates. Each point is coloured by the gene’s weight and subtype (PAM score) in the CRCA-786 centroids. Correlation co-efficient (Pearson’s *r*) value is shown.

nCounter® Max Analysis System (NanoString Technologies, Seattle, WA, USA) was used to perform the assay using either standard or modified (Elements chemistry) protocol as per the manufacturer’s instructions (Fig. 1a). For the standard protocol, custom CodeSets (pre-built capture probes tagged to biotin labels and reporter probes tagged to fluorescently colour coded molecular barcodes) for selected sets of genes were designed and built by NanoString Technologies. For the modified protocol, nCounter Elements™ TagSets (only capture and reporter tags; NanoString Technologies) and custom-designed target-specific oligonucleotide probe pairs (reporter/Probe A and capture/Probe B probes; from Integrated DNA Technologies, Inc., Leuven, Belgium) were obtained.

The TagSets consist of fluorescently-labelled specific Reporter Tags to resolve and count individual nucleic acid target sequences during data collection (Probe A), and biotinylated universal Capture Tags to capture the hybridised target nucleic acid sequence to the streptavidin-coated imaging surface (Probe B). Final hybridising concentration was 20 pM for Probe A and 100 pM for Probe B.

For both standard and modified protocols, 100 ng of total mRNA (20 ng/uL) from fresh-frozen or FFPE tissues was used. Hybridisation reactions were prepared according to manufacturers’ instructions for either 18 h at 65 °C using Standard CodeSets reagents for standard protocol or for 20 h at 67 °C using Elements™ TagSets reagents for the modified protocol. Hybridised samples were pipetted using the nCounter Prep Station and immobilised on to the sample cartridge for data quantification and collection using nCounter Digital Analyzer (NanoString Technologies). The nCounter Prep Station and Digital Analyzer together constitute the nCounter® Max Analysis System. For the PanCancer Progression Panel (NanoString Technologies), the standard protocol from the manufacturer was used. The processing and quality control steps are available in *Supplementary Information*.

### Assessing subtype weights in single samples using *idSample* tool

*idSample* employed support vector regression (SVR)^18^ based on CIBERSORT^19^ to train a model using reference data, such as centroids of values representing different subtypes - in this case, our published 786-gene signature-based PAM centroids^3^. The model was then used to predict the gene expression profiles of each sample from a test data set such that it provides weights for each subtype. Finally, the subtypes showing negative weights for each sample (meaning they were not represented in that sample) were set to zero, and the rest of those subtypes showing positive weights were normalised to compute the proportion of each subtype in each sample. Those samples with maximum proportion greater than 70% were selected as representing a single subtype.

### Selection of robust 38-gene signature using *intPredict* tool

We selected a robust set of genes that best predict the CRC subtypes with lowest misclassification error rate (MCR) using the samples selected by *idSample* and another in-house built machine-learning approach-based R package, *intPredict*. This tool employed a pipeline of different gene selection and class prediction methods to develop a robust gene classifier to predict subtypes by randomly splitting the original data set of samples into training and test data sets and executing the pipeline repeatedly 50 times (Monte Carlo cross-validation). Gene selection methods included prediction strength (PS)^20^, prediction analysis of microarrays (PAM)^21^ and between-within group sum of squares ratio (BW)^22^. Furthermore, the best performing gene set from the gene selection methods was identified using multiple class prediction methods such as random forest (RF)^23^, diagonal linear discriminant analysis (DLDA)^22^ and two support vector machine (SVM) approaches – linear and radial methods^18^. The MCR for each method was determined as follows,

1 $$MCR=\frac{1}{k}{\sum }_{i=1}^{k}{e}_{i}$$

where *k* is the number of test samples, and *e*_*i*_ is the misclassification of each test sample compared to known subtype. The gene set with the lowest median MCR of all the methods was chosen. For each gene set, a 95% credible interval on the median MCR was calculated.

### Assigning subtypes to samples

CRCA subtypes were assigned by performing Pearson correlation of gene-wise median-centred expression profiles for each sample with corresponding centroids for the subtypes. The subtype with the highest correlation was then assigned to that sample. Samples were marked as having “undetermined” subtype if the sample’s correlation with the subtype centroid had a value (Pearson’s *r*) ≤0.15, or if the correlation was high for multiple subtype centroids (Pearson’s *r* difference between first and second highest subtypes ≤0.06), in line with the published CMS classifier^13^.

CMS subtypes were determined from microarray or RNAseq data using the *CMSclassifier* R package (v1.0.0) and the *classifyCMS* function, using the single sample prediction (SSP) classifier^13^.

*Supplementary Information* contains further detailed information of all methods employed.

## Results and Discussion

### Analytical development and assessment of gene expression profiling using a reproducible assay

In order to develop an analytical method to classify CRC samples into subtypes, we initially developed a custom nCounter assay using a 50-gene panel (Supplementary Table S1d; 48 of which overlap with the original 786-gene signature; see *Materials and Methods*). Initially, we applied a standard protocol from the manufacturer (in which biotin labels and molecular barcodes are directly attached to the mRNA probes; Fig. 1a) and tested the performance of the custom nCounter assay using primary fresh frozen tumour RNA obtained from 22 CRCs from two different cohorts (Montpellier and OriGene; Supplementary Table S2a). These samples were an unbiased selection from the 34 total in these cohorts as an initial technical validation. Hierarchical clustering analysis using nCounter profiles clustered these 22 samples into different groups that potentially represent the different subtypes (Fig. 1b and Supplementary Fig. S1a).

Next, we evaluated if a “modified” protocol from NanoString Technologies (custom unique probes are attached to biotin labels and molecular barcodes separately; ~35% less expensive than standard protocol; Fig. 1a and Supplementary Table S1a) can deliver similar classification performance compared to the standard protocol-based assay. The samples (n = 22) from modified protocol clustered into potential subtypes in a similar fashion to the standard protocol (Supplementary Fig. S1b,c and Supplementary Table S2b).

Figure 1c shows the clustering of the same samples between the two protocols, for which gene expression was highly correlated (Pearson’s *r* = 0.90, p < 0.001; Fig. 1d and Supplementary Fig. S1d; data from protocols merged after gene-wise median centring). This demonstrates that we can successfully replicate results from the standard protocol using the modified protocol for a more cost-effective assay. When we performed subtyping of the matched samples from these different protocols, we found 100% (17/17) concordance between assigned subtypes for classifiable samples (refer to *Materials and Methods* for more details; Fig. 1e and Supplementary Table S3). Five samples were not classifiable from either or both the protocols. Hence, we adopted the modified protocol for our assay (Fig. 1a).

To test if our assay results are highly reproducible, we performed our assay on five of the above samples twice, in separate batches- a maximum of 40 weeks apart (Supplementary Table S2c,d). Figure 1f and Supplementary Fig. S1e shows the clustering of replicate samples together without batch effect and with high correlation of gene expression (Pearson’s *r* = 0.98, p < 0.001; Fig. 1g). This establishes the high reproducibility of our assay over non-negligible periods of time. Hence, in the future, we can use this assay to test the state of subtypes using matched pre- and post-treatment biopsies or resected materials.

### Analytical validation of gene expression assay using FFPE samples

**Figure 2:**
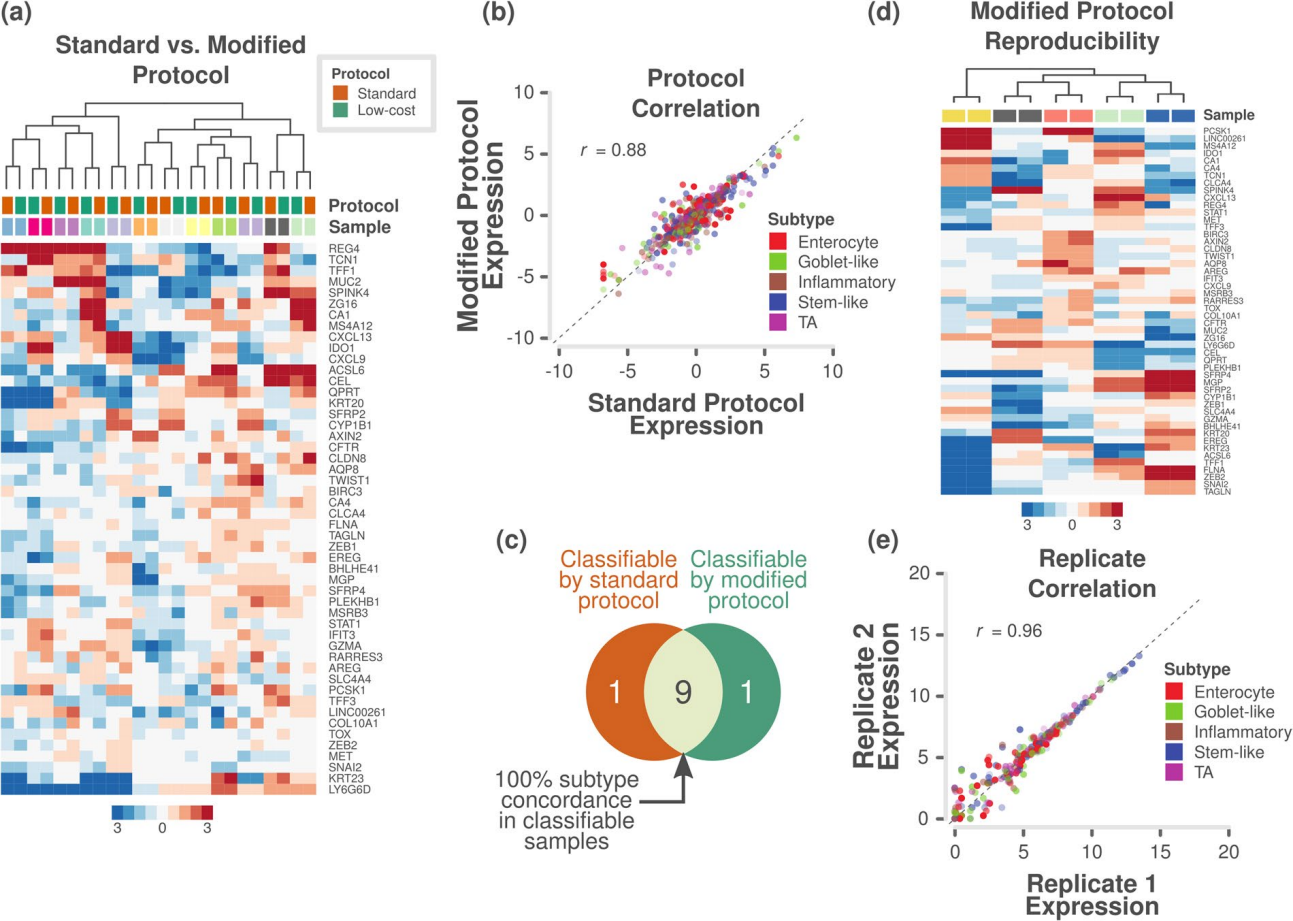
Assessment of protocols and reproducibility of reduced gene subtype-based nCounter assay in FFPE samples. (**a**) Heatmap of expression levels of the selected 48 subtype-specific genes (and 2 additional genes) for 12 patient samples from the RETRO-C cohort as measured on a custom nCounter panel using both standard and modified protocols (24 samples – 12 each from two protocols; median centred within protocols before clustering). (**b**) A scatter plot of gene expression measurements for 48 genes in 12 samples between the standard and modified protocols (median centred within protocols before correlation). Each point is coloured by the gene’s weight and subtype (PAM score) in the CRCA-786 centroids. Correlation co-efficient (Pearson’s *r*) value is shown. (**c**) Venn diagram indicating the number of samples that were classifiable by the standard and modified protocols, and the concordance between classifiable samples. (**d**) Heatmap of expression levels of the selected 48 subtype-specific genes (and 2 additional genes) from 5 technical duplicates assayed using modified protocol with a maximum interval of 13 weeks to show the reproducibility of the assay. (**e**) A scatter plot of gene expression measurements for 48 genes in 5 samples between technical duplicates. Each point is coloured by the gene’s weight and subtype (PAM score) in the CRCA-786 centroids. Correlation co-efficient (Pearson’s *r*) value is shown.

Although FFPE samples may contain low-abundance or highly degraded RNA, they also represent the most frequently available type of samples for diagnosis and biomarker assessment. Therefore, an efficient biomarker assay for FFPE samples is crucial for routine clinical application. Using the RETRO-C FFPE-preserved cohort of samples (see *Materials and Methods*), we again achieved successful clustering by sample rather than by protocol between the standard and modified protocols for 12 samples (Fig. 2a and Supplementary Fig. S2a–e and Supplementary Table S4a–d). Pearson’s correlation coefficient of gene expression between the protocols was 0.88 (p < 0.001; Fig. 2b). Further, subtyping showed 100% (9/9) concordance in subtypes between protocols (Fig. 2c and Supplementary Table S3). Three samples were not classifiable from either or both the protocols. Moreover, five pairs of technical replicates also showed highly reproducible results (Fig. 2d) with a Pearson’s correlation coefficient of 0.96 (p < 0.001; Fig. 2e and Supplementary Fig. S2f), similar to that for fresh frozen samples (Fig. 1g).

### Selection of robust gene set for subtyping

**Figure 3:**
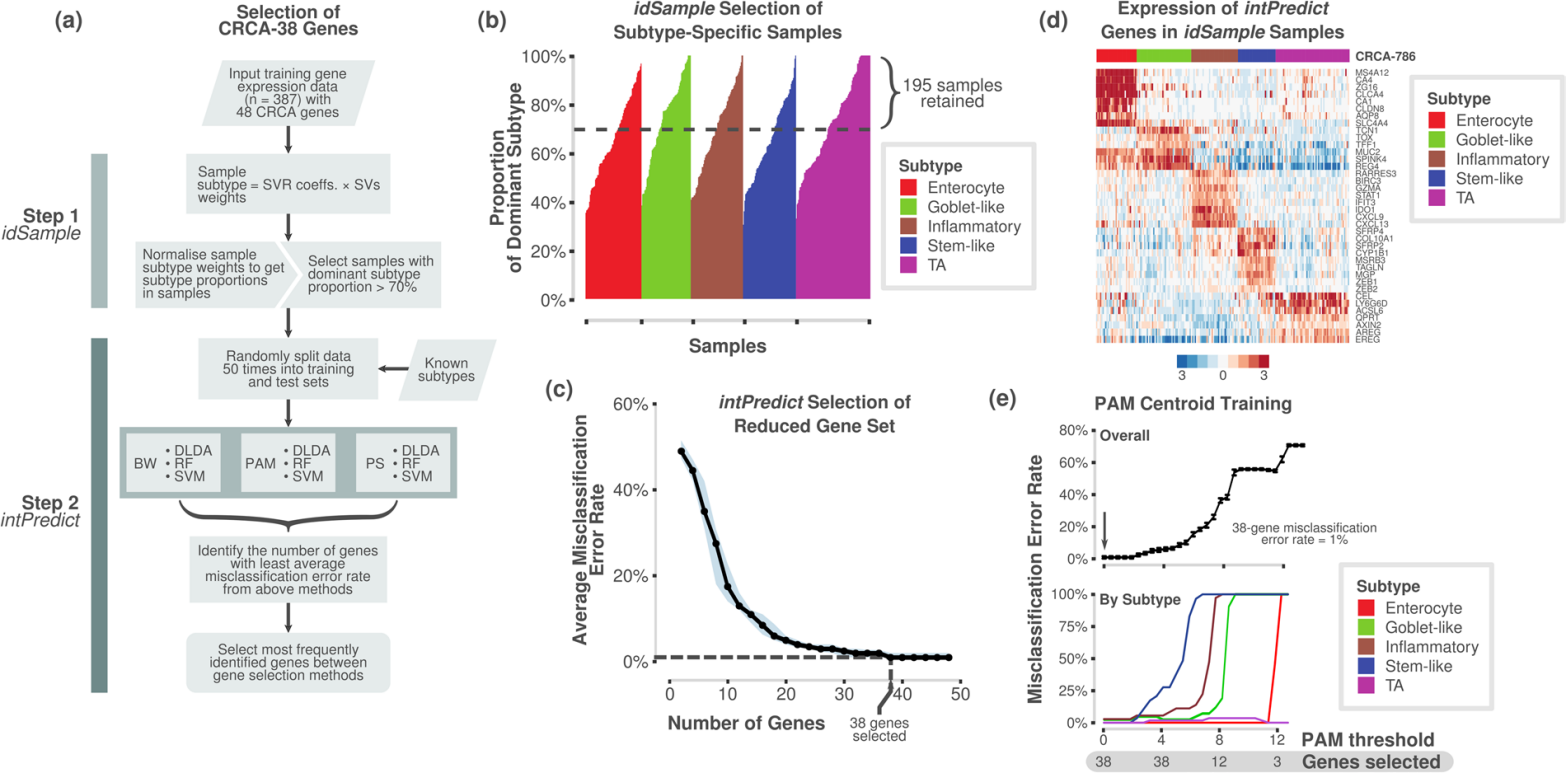
Selection of a robust 38-gene panel. (**a**) Overview of the process and pipelines used to select a robust gene set for the NanoCRCA assay using in-laboratory developed *idSample* and *intPredict* computational tools. (**b**) Bar plot showing the probability of a sample from our original published dataset (n = 387) belonging to a given subtype, as assessed using *idSample*. The dotted line represents a cut-off of 70% probability of a single subtype in each sample. (**c**) A line plot showing median MCR and number of genes as selected using the *intPredict* pipeline and samples selected in b) (n = 195). The light blue band shows the 95% credible interval of the median MCRs. (**d**) Heatmap showing the gene expression of the 38-gene panel selected by the *intPredict* pipeline in (**c**) in the 195 samples selected by *idSample* from b). The top bar shows the 786-gene signature-based subtype of the samples. (**e**) Line plots showing MCR using PAM at different numbers of genes for all the subtypes (upper) and individual subtypes (lower) for 195 samples from b). PS - prediction strength, PAM - prediction analysis of microarrays, BW - between-within group sum of squares ratio, RF - random forest, DLDA - diagonal linear discriminant analysis, SVM - support vector machine, SVR - support vector regression (SVR), and SV – support vector.

Successful clinical biomarker assays should be able to classify samples into subtypes with high confidence, and this requires a robust set of genes. Hence, we tested the accuracy of our selected 48 genes (from the 786-gene signature) using two in-laboratory developed bioinformatics tools – *idSample* and *intPredict* (Fig. 3a; *Materials and Methods*). Since mixed subtype samples comprising more than one subtype are present in CRC^13^, we selected only the samples from our published training dataset (n = 387)^3^ that showed at least 70% probability of belonging to a single tumour subtype (similar to the 70% tumour cellularity applied in many transcriptomic studies; n = 195; Fig. 3b; Supplementary Table S5a) using our *idSample* tool (*see Materials and Methods*). Furthermore, the *intPredict* tool, which contains a pipeline of supervised gene selection (PS^20^; PAM^21^; and BW^22^) and class prediction methods (RF^23^; DLDA^22^; and SVM^18^), was used to identify 38 robust genes with the lowest misclassification error rate (MCR, 1%; Fig. 3c,d; Supplementary Table S5a,b) out of the 48 CRCA-786 genes on the panel. Briefly, this was achieved by repeatedly splitting the dataset into test and training sets, to which each combination of gene selection and class prediction methods was applied, and the MCR calculated. Further details of this procedure can be found in *Materials and Methods*. Subsequently, the gene set with the lowest median MCR was chosen. In order to then classify new samples into the CRC subtypes using the selected 38-gene panel, we derived new 38-gene centroids using the *idSample*-selected samples (named *CRCA-38* classifier; using PAM methodology; Fig. 3e) having only 1% MCR.

### Validation of NanoCRCA in fresh frozen samples

**Figure 4:**
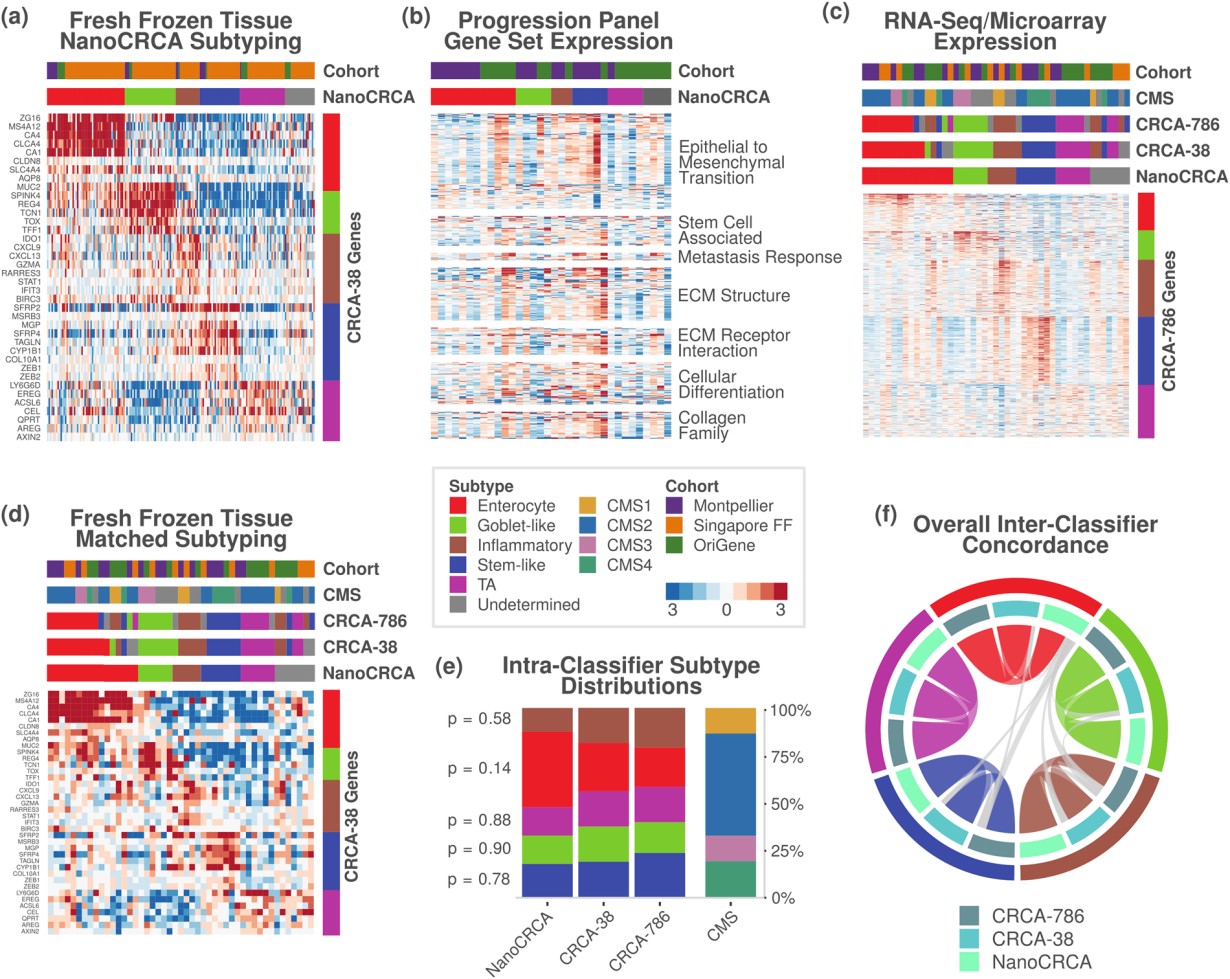
NanoCRCA subtyping, pathway and 786-gene signature analysis with subtype stability. (**a**) Heatmap showing the expression of the 38-gene-panel as measured using the NanoCRCA assay in the three fresh frozen cohorts (n = 179). From top to bottom, the upper bars indicate the cohort and the NanoCRCA subtypes as determined by nCounter profiles. The right-hand vertical bar indicates the subtype association of each gene. (**b**) Heatmap of nCounter PanCancer Progression Panel-based gene expression profiles from the Montpellier and OriGene cohorts of samples (n = 34). Upper bars are as in a). Genes are grouped according to functional annotations provided by NanoString Technologies. (**c**,**d**) Heatmap of c) RNAseq/microarray gene expression profiles and d) NanoCRCA gene expression profiles from samples from all the three cohorts having matched data (n = 47). From top to bottom, the upper bars indicate the cohort, the CMS subtype, the CRCA-786 and CRCA-38 subtypes as determined by microarray/RNAseq profiles, and the NanoCRCA subtypes as determined by nCounter profiles. The right-hand vertical bar indicates the subtype association of each gene. (**e**) Distribution of subtypes according to each classifier. Samples that were of undetermined subtype were excluded for each classifier (NanoCRCA n = 40; CRCA-38 n = 43; CRCA-786 n = 43; CMS n = 37). P-values resulting from statistical tests of proportion for each subtype between the three CRCA classifiers are shown on the left-hand side. (**f**) Chord plot illustrating the tendency of samples to be classified as the same subtype between the three assays. Samples from all three cohorts which had no undetermined subtype calls were included (n = 34). Each arc connects the classification of a sample in two different assays, and each sample is represented by three arcs (connecting NanoCRCA, CRCA-38 and CRCA-786 subtypes). Samples with the same subtype in all three assays are coloured by that subtype. Samples that had discordant classification between the assays are coloured grey (4/34 samples).

To determine if these assays could successfully stratify patient samples into CRCA subtypes, we applied our “NanoCRCA” assay (utilising the modified protocol and 38-gene signature from the CRCA-38 classifier) to fresh frozen CRC samples (n = 179; combined samples from the Montpellier, Singapore FF and OriGene cohorts). Subtypes were determined by the correlation of gene expression profiles with the CRCA-38 centroids. All five subtypes were identified by the assay (with 89% (159/179) of the samples being classifiable) and demonstrated distinct patterns of gene expression (Fig. 4a). A small proportion of samples (11%; 20/179) were found to be of undetermined subtype (in that they cannot be classified into any of the five subtypes), either because they have mixed subtype or are of poor quality, which were identified using correlation coefficient cut-offs as discussed in our CMS study^13^ (see *Materials and Methods*; Fig. 4a; Supplementary Table S6). Overall, our NanoCRCA assay was successfully applied in fresh frozen samples.

### Molecular characteristics of the NanoCRCA subtypes

We further confirmed the molecular characteristics of the subtypes from NanoCRCA, especially stem-like samples, which showed increased expression of genes associated with EMT, stem cells, metastatic response, extracellular matrix (ECM) structure and receptor interaction, cellular differentiation, the collagen family and others (Fig. 4b, Supplementary Fig. S3a–d and Supplementary Table S7a,b). This was done by profiling 34 fresh frozen samples from the Montpellier and OriGene cohorts using NanoString Technologies’ PanCancer Progression Panel. In addition, the expression of the 786-gene signature as measured by RNAseq or microarrays is shown in Fig. 4c for samples with matched data (n = 47; Montpellier, Singapore FF and OriGene cohorts; Supplementary Fig. S3e,f), alongside the NanoCRCA subtypes of the samples. Overall, these analyses demonstrate that the subtypes identified by the NanoCRCA assay represent the published molecular characteristics of these subtypes^3^ in 179 samples from three independent cohorts.

### Concordance and distribution of subtypes between CRCA classifiers, platforms and CMS subtypes

We sought to assess if subtyping using the NanoCRCA assay (with the modified protocol and 38-gene classifier) mirrored the results of subtyping using CMS subtypes and platforms such as microarrays and RNAseq. Although these platforms are highly multiplexed, they can be expensive and have long sample and data processing time (between 2 and 12 months; Supplementary Table S1a). Matched microarray or RNAseq data for the fresh frozen Montpellier, Singapore FF and OriGene cohorts were generated or obtained from public repositories (see *Materials and Methods;* n = 47). Subtypes from CRCA-38 (using the same centroids as utilised to classify samples using NanoCRCA), the original CRCA-786 and CMS were determined on these platforms. Figure 4d shows the expression of the CRCA-38 classifier genes in these samples as measured by the NanoCRCA assay, alongside their subtypes as assigned by the other classifiers or platforms. The platform and gene set differences did not significantly bias the distribution of subtypes assigned to the samples (n = 47) across the three CRCA assays (p > 0.05; proportion tests; Fig. 4e and Supplementary Table S8a–e). The trend (although not statistically significant) towards slightly higher numbers of enterocyte samples using NanoCRCA needs to be assessed fully in a larger cohort, and could be due to normal contamination in fresh frozen samples. However, overall concordance between platforms was good (Fig. 4f; n = 34; Supplementary Table S6), with 88% (30/34) of samples showing the same subtype across all 3 classifiers/assays.

### Assessment of CRC subtypes from NanoCRCA assay and other platforms and classifiers in the Montpellier cohort of fresh frozen samples

**Figure 5:**
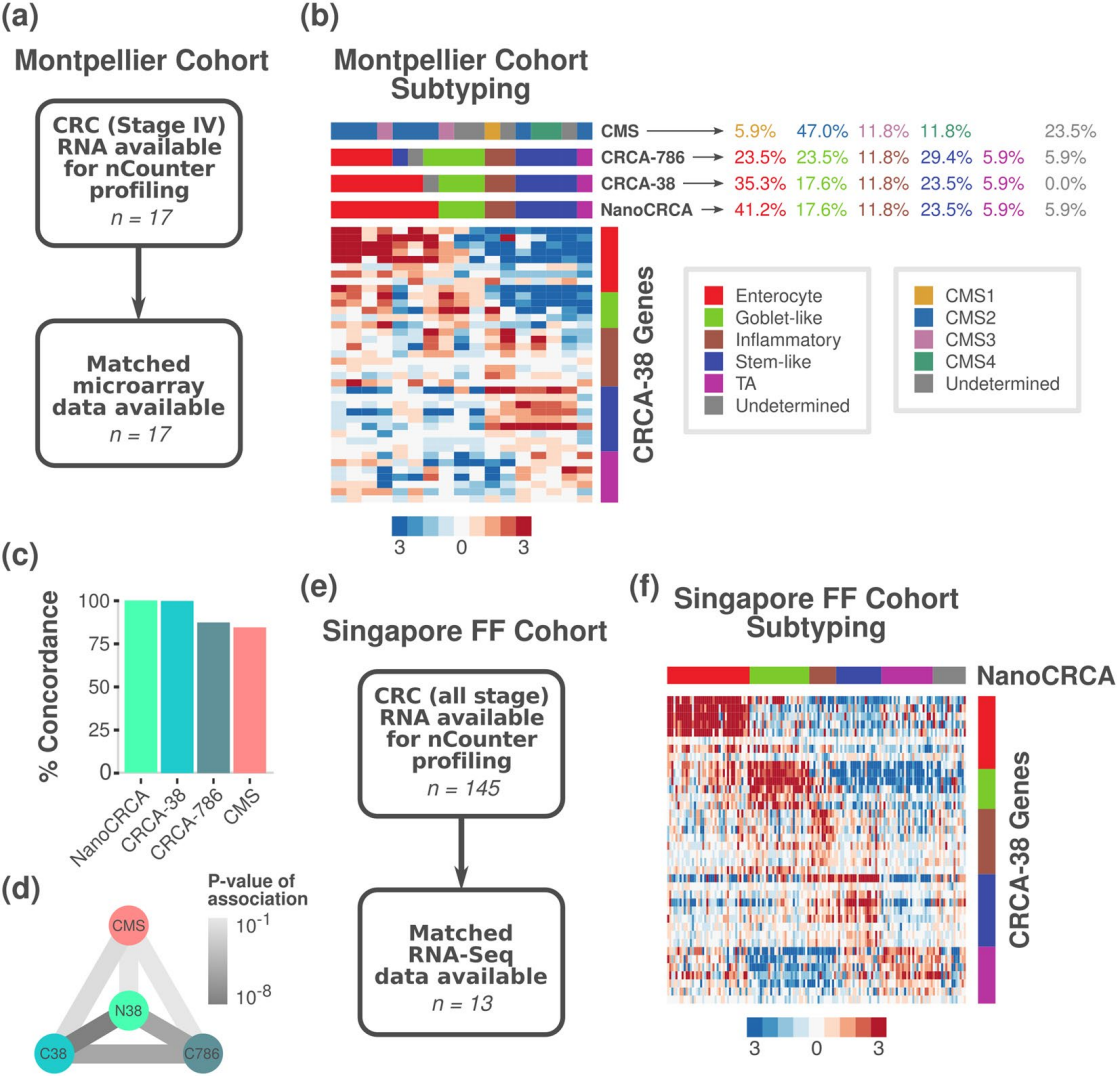
Montpellier cohort: NanoCRCA assay, its comparison with other platforms and the CMS classifier; and Singapore FF cohort: NanoCRCA assay. (**a**) A summary of the Montpellier cohort showing patient characteristics, sample size and available microarray data. (**b**) Heatmap showing the expression of the 38-gene-panel in the Montpellier cohort as measured using NanoCRCA assay (n = 17). From top to bottom, the upper bars indicate the CMS, CRCA-786 and CRCA-38 subtypes as determined by microarray profiles, and the NanoCRCA subtypes as determined by nCounter profiles. The right-hand vertical bar indicates the subtype association of each gene. The percentage of samples falling into each CMS and CRCA subtype is shown on the right. (**c**,**d**) Comparisons between NanoCRCA and microarray-based classifications CRCA-38, CRCA-786 and CMS showing c) percent concordance to NanoCRCA and d) statistical significance (Fisher’s exact test) in the Montpellier cohort. (**e**) A summary of the Singapore FF cohort showing patient characteristics and sample size. (**f**) Heatmap showing the expression of the 38-gene panel in the Singapore FF cohort as measured using NanoCRCA assay (n = 145). The subtypes as assigned using the NanoCRCA assay are shown on the top bar. The right-hand vertical bar indicates the subtype association of each gene. Subtype colours are as in b).

In order to understand the utility of stratifying CRC samples using NanoCRCA, we further analysed our Montpellier cohort of 17 primary tumours from stage IV patients^4,6^ (*Materials and Methods*; Fig. 5a and Supplementary Fig. S4a,b; Supplementary Table S9a,b). All the CRCA subtypes were present in this cohort, and all samples were successfully classified, with none showing undetermined subtype (Fig. 5b). We observed a non-uniform distribution of the subtypes, with enterocyte comprising 41.2% (7/17), followed by stem-like (23.5%; 4/17), goblet-like (17.6%; 3/17) and inflammatory (11.8%; 2/17) subtypes. The TA subtype was lower in frequency in this cohort of samples (5.9%; 1/17).

We additionally compared our NanoCRCA assay with the microarray-based CMS subtypes in this cohort. We classified the Montpellier cohort of 17 microarray gene expression profiles into CMS subtypes using the published CMS classifier^13^. We successfully classified the samples into all of the CMS subtypes: 47.0% (8/17) were CMS2 (generally associated with enterocyte and TA); 11.8% (2/17) each of CMS3 (goblet-like) and CMS4 (stem-like); and 5.9% (1/17) of CMS1 (inflammatory). However, we found 23.5% (4/17) samples with undetermined subtype (defined as “mixed” samples in the original publication^13^). Again, the NanoCRCA assay showed good and significant concordance (84.6% (11/13); Fisher’s exact test p = 0.01) with the CMS classifier excluding the undetermined samples (Fig. 5c,d and Supplementary Table S8c). Further validation is required to test if NanoCRCA assay can consistently predict CMS subtypes (as the CMS classification was partly derived from the CRCA classification) with reasonable concordance, which is beyond the scope of the current study. However, as the two classification systems are highly concordant, but not directly equivalent, they might perform slightly differently in both the research and clinical settings (in particular, the enterocyte and TA subtypes in the CRCA classifier have different therapy responses, and are combined into a single CMS2 subtype in CMS classifier^3,13,14^). Two separate assays (one for CMS, yet to be developed, and NanoCRCA for CRCA subtypes) may be more appropriate if translation of CRC subtypes into the clinic is to be achieved.

We also compared the performance of the NanoCRCA assay against classification using Affymetrix Human Genome U133 Plus 2.0 (HG-U133 Plus2) microarray profiles and the CRCA-38 classifier in this cohort (Fig. 5b and Supplementary Fig. S4c). We classified the samples’ microarray profiles into all the five subtypes with 35.3% (6/17) as enterocyte, 23.5% (4/17) as stem-like, 17.6% (3/17) as goblet-like, 11.8% (2/17) as inflammatory and 5.9% (1/17) as TA (Supplementary Fig. S4d). Only one sample was defined as having undetermined subtype (5.9%; 1/17), expressing both inflammatory and enterocyte genes. Overall, the NanoCRCA assay showed perfect concordance with the microarray-based CRCA-38 classification after excluding samples with undetermined classification (Fig. 5c,d).

Similarly, we compared the CRCA-786 classification using microarray data to the results of NanoCRCA. The 786-gene signature-based classification yielded 29.4% (5/17) stem-like, 23.5% (4/17) each enterocyte and goblet-like, 11.8% (2/17) inflammatory and 5.9% (1/17) TA samples (Supplementary Fig. S4e). There was again one undetermined sample (5.9% (1/17); Fig. 5b and Supplementary Fig. S4e). Despite the different number of genes profiled on the different platforms, the NanoCRCA assay showed 87.5% concordance with the microarray-based CRCA-786 subtypes (14/16; Fisher’s exact test p < 0.0001; Fig. 5c,d; Supplementary Table S8c). In sum, the three assays performed using two different platforms identified all the five subtypes, and the NanoCRCA assay predicted subtypes highly consistent with the microarray platform.

### Assessment of CRC subtypes from NanoCRCA assay in the Singapore and OriGene cohorts of fresh frozen samples

When we went on to examine the subtypes identified in our largest cohort of fresh frozen samples originating from Singapore (Fig. 5e,f and Supplementary Fig. S5a,b; Supplementary Table S9c,d), we found a more uniform distribution of subtypes, perhaps due to the vastly increased sample size compared to other cohorts (n = 145). The enterocyte subtype was still the most prevalent (27.6%; 40/145), followed by goblet-like (20.0%; 29/145), TA (17.2%; 25/145), stem-like (15.2%; 22/145) and inflammatory (9.0%; 13/145). There were 11.0% (16/145) undetermined samples. While the number of samples with matched RNAseq data was low (n = 13; Supplementary Fig. S5c), we found a significant association between the subtype calls from NanoCRCA and RNAseq-based CRCA-38, and 77.8% concordance (7/9; Fisher’s exact test p = 0.02; Supplementary Table S8d and Supplementary Fig. S5d,e). Between NanoCRCA and RNAseq-based CRCA-786, the association did not reach significance (p = 0.11; Supplementary Table S8d and Supplementary Fig. S5d,e), but concordance was high at 86% (6/7) indicating this may be due to low sample number. Overall, this large cohort illustrates that the NanoCRCA assay can reliably call subtypes in fresh frozen samples concordant with the RNAseq platform. Similarly, the OriGene cohort showed significant concordance between NanoCRCA and microarray subtyping (Supplementary Fig. S6a–d; Supplementary Tables S8e and S9e,f).

### Evaluation of assay in matched fresh frozen and FFPE tissues

**Figure 6:**
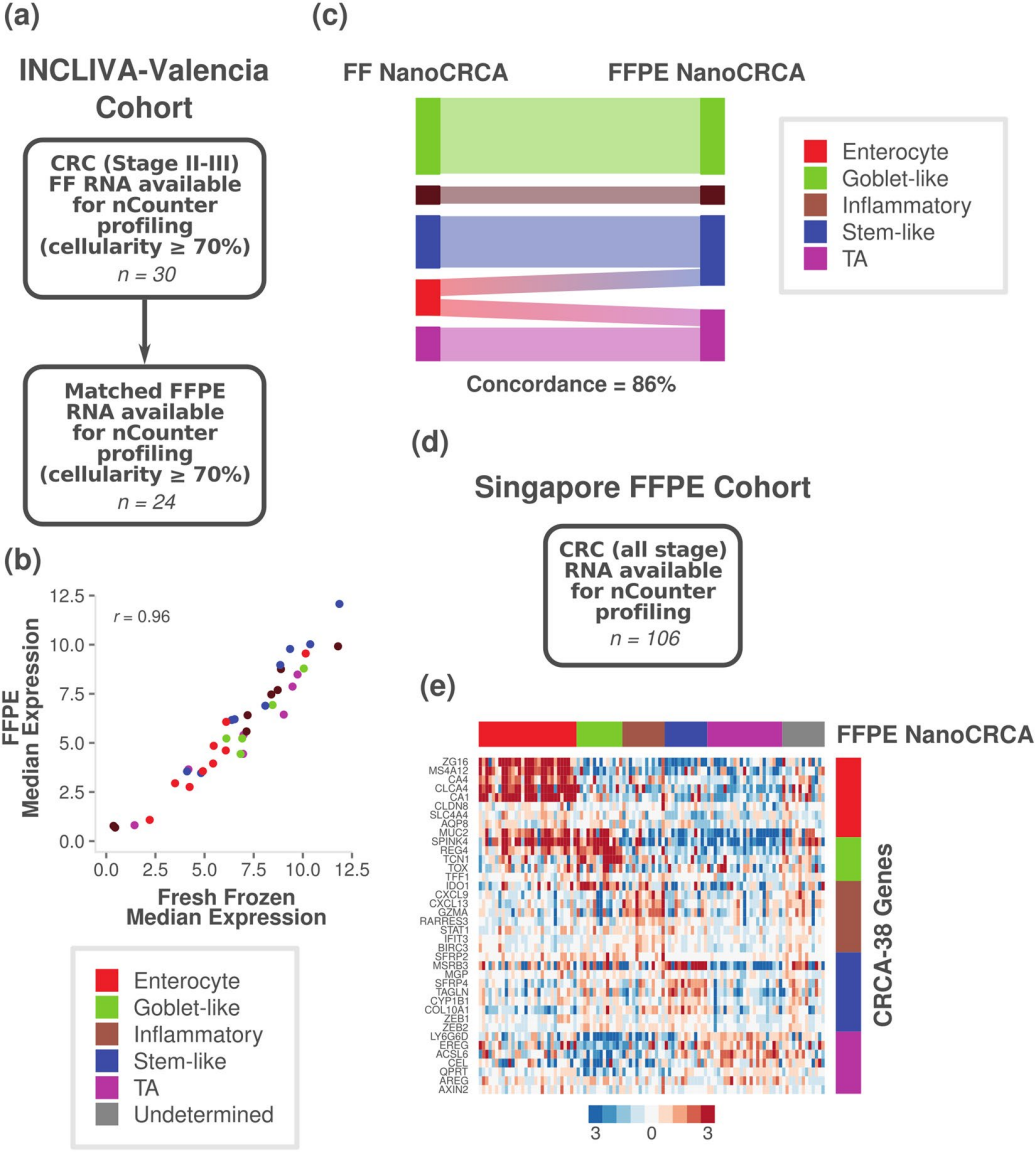
Translation of assay via matched fresh frozen and FFPE tissue. (**a**) A summary of the INCLIVA-Valencia cohort, showing patient characteristics and sample size. (**b**) A scatter plot showing median expression of the 38 genes in samples with tumour cellularity ≥70% in both fresh frozen and FFPE tissues (n = 24). Colours indicated each gene’s association with the subtypes. (**c**) Alluvial diagram showing the subtype classification of matched fresh frozen and FFPE tissues for cellularity-selected samples (excluding undetermined samples; n = 14). (**d**) A summary of the Singapore FFPE cohort, showing patient characteristics and sample size. (**e**) A heatmap showing the expression of the 38-gene panel in Singapore FFPE samples as measured using the NanoCRCA assay (n = 106). The subtypes as assigned using the NanoCRCA assay are shown on the top bar. The right-hand vertical bar indicates the subtype association of each gene. Subtype colours are as in b).

Finally, we sought to validate the NanoCRCA assay’s applicability to FFPE samples by utilising our INCLIVA-Valencia cohort of 24 stage II-III patients having matched fresh frozen and macrodissected FFPE CRC samples (Fig. 6a and Supplementary Fig. S7a–d; Supplementary Table S9g–j), and tumour cellularity of ≥70% for both (see *Supplementary Information*). In addition to our prior finding of high reproducibility of the assay in FFPE samples (Fig. 2d,e), we found a high correlation between median gene expression measured using fresh frozen and FFPE tissues (Pearson’s *r* = 0.96, p < 0.0001, n = 24; Fig. 6b).

Figure 6c and Supplementary Fig. S7e show the assigned subtypes and gene expression in the matched samples, respectively. All the samples, excluding enterocyte samples, showed the same subtype identity between fresh frozen and FFPE tissues. However, enterocyte subtype samples (n = 2) from fresh frozen tissues were classified as TA and stem-like in FFPE tissues (Fig. 6c). This can be explained partially by the similarity between enterocyte and TA subtypes that combined together to form the CMS2 subtype in the CMS classification^13^. Further investigation is required to determine if this is a systematic effect, perhaps affected by tumour cellularity, by gathering a larger cohort of matched fresh frozen and FFPE tissues.

### Validation of NanoCRCA assay using an additional FFPE cohort of samples

Finally, we validated our NanoCRCA assay using an additional 106 FFPE samples from the Singapore FFPE cohort (Fig. 6d,e and Supplementary Fig. S8a,b; Supplementary Table S9k,l), separate to those analysed from the Singapore FF cohort. Samples could be classified into all 5 CRCA subtypes, and exhibited characteristic gene expression of the subtypes (Fig. 6e). The majority of samples fell into the enterocyte subtype (28.3%; 30/106), followed by the TA (21.6%; 23/106), goblet-like (13.2%; 14/106), stem-like (12.3%; 13/106) and inflammatory (12.3%; 13/106) subtypes. 13 samples were unclassifiable (12.3%). This clearly suggests that the NanoCRCA assay is applicable to large cohorts of the FFPE samples that are ubiquitously available for diagnostics.

## Conclusion

In summary, we developed and analytically validated our NanoCRCA biomarker assay based on a refined 38-gene classifier, and classified CRC samples into molecular subtypes, along with undetermined samples. Subtype prediction by the NanoCRCA assay is highly concordant with more multiplexed platforms, and predicted subtypes show the expected association with the CMS subtypes. In addition, the same 38-gene signature can be applied to subtype both whole-transcriptome data (microarrays or RNAseq) and nCounter data, allowing for equivalent interpretation of results from both of these platform types. However, care must be taken to ensure sufficient cellularity of the samples and careful selection of the type of samples (fresh frozen vs. FFPE) prior to subtyping to avoid misclassification. Most importantly, our NanoCRCA is highly applicable to clinically available FFPE samples, potentially more so than even fresh frozen samples. Since multiple CRC clinical trials require low-cost, reproducible and rapid clinically implementable assays to prospectively validate CRC subtypes for subtype-specific studies, our NanoCRCA assay may potentially facilitate this process in the clinic using FFPE samples.

## Supplementary information

Supplementary Information & Figures

Supplementary Table S1

Supplementary Table S2

Supplementary Table S3

Supplementary Table S4

Supplementary Table S5

Supplementary Table S6

Supplementary Table S7

Supplementary Table S8

Supplementary Table S9

## Data Availability

Previously published GEO Omnibus data sets were analysed for gene set selection (GSE14333 and GSE13294) and microarray-based subtyping of the Montpellier cohort (GSE62080). nCounter data for all cohorts (GSE101479 – standard protocol and GSE101481 – modified protocol) and microarray/RNAseq data for OriGene (GSE101472) and Singapore (GSE101588) cohorts are deposited under the SuperSeries with accession number GSE101651. The rest of the data are part of the supplementary Tables.
