# Supplementary material for: Analytical Validation of Multiplex Biomarker Assay to Stratify Colorectal Cancer into Molecular Subtypes
Source: Sci Rep. 2019 May 21;9:7665. doi: 10.1038/s41598-019-43492-0 (PMC6529539; doi:10.1038/s41598-019-43492-0)
Supplement: Supplementary file 1 — Supplementary Information & Figures [file 41598_2019_43492_MOESM1_ESM.pdf]

Supplementary Information

Analytical Validation of Multiplex Biomarker Assay to Stratify Colorectal Cancer into Molecular Subtypes

Chanthirika Ragulan[1,2,†], Katherine Eason[1,†], Elisa Fontana[1,2,†], Gift Nyamundanda[1,2,†], Noelia Tarazona[3], Yatish Patil[1,2], Pawan Poudel[1], Rita T. Lawlor[4,5], Maguy Del Rio[6], Koo Si-Lin[7], Tan Wah Siew[8], Francesco Sclafani[9], Ruwaida Begum[9], Larissa S. Teixeira Mendes[2], Pierre Martineau[6], Aldo Scarpa[4,5], Andrés Cervantes[3], Iain Beehuat Tan[8,10,11], David Cunningham[2,9] and Anguraj Sadanandam[1,2,*]

1. Division of Molecular Pathology, The Institute of Cancer Research, London, United Kingdom
2. Centre for Molecular Pathology, The Royal Marsden NHS Foundation Trust, London, United Kingdom
3. CIBERONC, Department of Medical Oncology, Biomedical Research Institute INCLIVA, University of Valencia, Valencia, Spain
4. ARC-Net Centre for Applied Research on Cancer, University and Hospital Trust of Verona, Verona, Italy
5. Department of Pathology and Diagnostics, University and Hospital Trust of Verona, Verona, Italy
6. Institut de Recherche en Cancérologie de Montpellier, Institut National de la Santé et de la Recherche Médicale, U896, Université Montpellier, Centre Régional de Lutte contre le Cancer Val d'Aurelle Paul Lamarque, Montpellier, France.
7. National Cancer Centre Singapore, Singapore
8. Singapore General Hospital, Singapore

[9.] Department of Medicine, The Royal Marsden NHS Foundation Trust, London, United Kingdom

[10.] Genome Institute of Singapore, Singapore

[11.] Duke-NUS Medical School, Singapore

[†] These authors contributed equally as first authors

[*] **Correspondence to**

Anguraj Sadanandam, Ph.D., Systems and Precision Cancer Medicine Team, Institute of Cancer Research (ICR), 15 Cotswold Road, Sutton, Surrey, SM2 5NG, United Kingdom; Email: anguraj.sadanandam@icr.ac.uk

Tel: +44 (0) 20 8915 6631

Running title: Colorectal Cancer Subtype Biomarker Assay

Keywords: colorectal cancer, subtype diagnostic assay, biomarker prediction, multiplex biomarker assay, nCounter platform, FFPE, fresh frozen

Supplementary Methods and Materials

RNA isolation and quality control

For the Singapore fresh frozen (FF) cohort, 15 mg of tissues were homogenised using 1mL of Invitrogen™ TRIzol™ (Thermo Fisher Scientific, Singapore) with gentleMACS™ M tube using gentleMACS™ Octo Dissociator (Miltenyi Biotec, Singapore) as per the manufacturer's instructions. The total RNA from homogenised tissues was extracted using QIAGEN RNeasy Mini Kit (QIAGEN, Singapore), following the manufacturer's protocol.

For fresh frozen samples from INCLIVA-Valencia cohort, tumour blocks with high tumour content were selected by a pathologist. The total RNA from homogenised tissues was extracted using Ambion RecoverAll™ kit (Life Technologies, CA, USA) as per manufacturers' instructions.

For formalin fixed paraffin embedded (FFPE) samples from INCLIVA-Valencia and RETRO-C cohorts, areas with high tumour content were marked on an H&E slide and macrodissected in 7-10 unstained slides (7-10 µm) using a xylene based deparaffinization method. The total RNA was extracted using the Ambion RecoverAll™ protocol as per manufacturer's instructions (Life Technologies). FFPE samples from Singapore FFPE cohort were isolated using Qiagen RNeasy FFPE kit (Qiagen).

All samples were quantified using NanoDrop 2000 Spectrophotometer (ThermoFisher Scientific, East Grinstead, UK). Certain samples were also assessed using Agilent RNA 6000 Nano Kit (Agilent, Santa Clara, CA, USA) where required.

Processing and quality control of nCounter data

Data quality from NanoString assay was checked and data normalization was performed using nSolver analysis software v3.0 (NanoString Technologies). Firstly, counts were corrected to background noise using geometric means of 8 negative control probes followed by the correction using geometric means of 6 internal positive control spike-ins in each lane/sample to correct potential sources of variations across the samples. These negative and positive probes were built-in in both standard and modified protocols. Only those housekeeping genes with raw molecular counts greater than 50 and those selected by geNorm algorithm (part of the nSolver analysis software) were retained for further analysis. Variations due to RNA input volume were corrected by normalising to the expression of geNorm selected housekeeping genes. The normalised final count data were $\log_2$ transformed for further analysis. Data generated from PanCancer Progression were also analysed for biological pathways using the nCounter Advanced Analysis plugin (v1.0.84) for nSolver analysis software.

Comparison of standard and modified protocols

For comparison of standard and modified protocols, housekeeping genes that were common between these two datasets were used for normalisation. Data from standard and modified protocols were row (gene)-median centred across samples separately, before being combined to perform hierarchical clustering or Pearson correlation analysis.

Generation and quality control of microarray data

For the OriGene cohort, 100 ng of total RNA was used for first strand cDNA synthesis and labelled according to the manufacturer's protocol. Labelled single stranded cDNA was hybridised in GeneChip Human Transcriptome Array (HTA) 2.0 (Affymetrix, High Wycombe, UK) then arrays were washed (Gene Chip Fluidics station 450) and scanned (Gene Chip Scanner).

Generation, quality control and analysis of RNA-Seq data

RNAseq libraries were prepared using TruSeq Stranded mRNA Library Prep Kit (Illumina, Singapore). Libraries were quality controlled using KAPA qPCR (Roche, Singapore) and Agilent Bioanalyzer, before pooling and sequencing on the Illumina HiSeq (Illumina) to a median of 22 million paired reads per sample. Fastq files were checked for reads counts for paired end reads and read quality using fastqc (v0.11.4)[1]. All 17 samples had mean Phred score greater than 34. Mapping quality was checked using RSEM (v1.2.22)[2] - samtools-flagstat (1.3.1)[3] using reference transcriptome (GRCh37). RNA quality (ribosomal, coding, intronic and intergenic) was checked using the CollectRnaSeqMetrics function of picard (v2.1.0)[4] on mapping to reference genome (GRCh37). Transcripts per million (TPM) values were calculated using RSEM, genes with <20% missing values were retained and $\log_2$(TPM+1) transformed.

Subtype concordance and significance

Subtype concordance between two different platforms was calculated as the percentage of samples that showed the same subtype in both (not including undetermined samples). Subtypes were deemed concordant between CRCA and CMS subtypes based on the following association: CMS1≈Inflammatory; CMS2≈Enterocyte and TA; CMS3≈Goblet-like; CMS4≈Stem-like[5]. Fisher's exact test or chi-squared test were used as appropriate to assess significance of associations; proportion tests[6] were used to compare distributions of subtypes between assays.

Batch effect assessment and merging of microarray/RNAseq datasets

Batch effects for any particular platform or cross-platforms were assessed using principal component analysis (PCA). Microarray (Montpellier and OriGene cohorts) and RNAseq (Singapore cohort) $\log_2$ normalised data (n=51) were merged after correcting batch effects using the ComBat[7] (*sva* R package v3.22.0) tool, for the purpose of heatmap visualisation only in Figure 4c (not subtyping), to remove any platform-specific effects. Supplementary Figure S3e shows the results of PCA before and after correcting batch effects in these pooled cohorts.

Clustering and heatmaps of samples' gene expression

All sample clustering was performed using Euclidean distance and complete linkage as implemented in the hclust and dist functions of the R package *stats* (v3.3.2). Arc plots were generated using the *circlize* package (v0.4.0). Heatmaps were plotted from gene-wise median-centred expression data thresholded to [-3, 3] using the *heatmap.plus* package (v1.3).

R packages and availability of our packages

R package *e1071* (v1.6-8)[8] was utilised for both SVM methods; *randomForest* (v4.6-12)[9] for RF; *sma* (v0.5.17)[10] for BW and DLDA; *pamr* (v1.55)[11] for PAM; and *stats* for tests of proportions[6]. An R package *intPredict* and other tools are available at GitHub https://github.com/syspremed/.

Supplementary Figure Legends

Supplementary Figure S1. PCA and clustering analysis of standard and modified protocol assays for fresh frozen samples. a-b. PCA of the combined Montpellier and OriGene cohorts of samples (n=22) assayed using the a) standard protocol and b) modified protocol. **c.** Heatmap of expression levels of selected 50 (48 CRCA + 2 other) genes for these samples (n=22) as measured on a custom nCounter panel using the modified protocol. The top bar shows the cohort each sample originated from (colours as in legend provided). **d-e.** PCA analysis of d) combined standard and modified protocols (after median centring of each dataset separately by protocol; n=22) and e) median-centred data of two technical replicates performed at an interval with median 40 weeks using the modified protocol (n=5).

Supplementary Figure S2. PCA and clustering analysis of standard and modified protocol assays for FFPE samples. a. PCA analysis of the RETRO-C cohort of samples assayed using the standard protocol (n=12). **b.** Heatmap of expression levels of selected 50 (48 CRCA + 2 other) genes for these samples as measured on a custom nCounter panel using standard protocol. The top bar shows the cohort each sample originated from (colours as in legend; n=12). **c.** PCA analysis of the RETRO-C cohort of samples assayed using the modified protocol (n=12). **d.** Heatmap of expression levels of selected 50 (48 CRCA + 2 other) genes for these samples as measured on a custom nCounter panel using the modified protocol (n=12). **e-f.** PCA analysis of e) combined standard and modified protocols (after median centring of each dataset separately by protocol; n=12) and f) two technical replicates performed with the modified protocol (n=5). Cartridges represent samples assayed at different time points/batches.

Supplementary Figure S3. PCA and heatmaps of nCounter PanCancer Progression Panel for Montpellier and OriGene cohorts. a. PCA analysis of nCounter PanCancer Progression panel data showing the distribution of samples run in three batches from the Montpellier and OriGene cohorts (n=34). **b.** Heatmap of expression of all 740 genes measured on this nCounter PanCancer Progression panel (n=34). **c-d.** Heatmaps showing the expression of all genes from different pathways in each PanCancer Progression Panel gene set shown in Figure 4b (n=34). Top bars in b-d) show the NanoCRCA, CRCA-38, CRCA-786 and CMS subtype for each sample and the batch in which that sample was processed. Colours for batch/cartridge are as in the legend. **e-f.** PCA analysis of merged Montpellier, Singapore FF and OriGene cohorts (n=51) e) before (left panel) and f) after (right panel) batch correction using COMBAT.

Supplementary Figure S4. PCA and clustering of nCounter assay from the Montpellier cohort. a. PCA analysis of NanoCRCA data for this cohort showing the distribution of samples run in two batches (n=17). **b.** Heatmap of all 50 (48 CRCA + 2 other) genes measured on the nCounter platform (n=17). Top bars show the subtypes assigned by the four classifiers - NanoCRCA, CRCA-38, CRCA-786 and CMS classifications, and the batch in which each sample was processed. Colours for batch/cartridge are provided. **c.** PCA analysis of microarray data (GSE62080) for this cohort (n=17). **d-e.** Distribution of subtypes for this cohort according to the d) CRCA-38 and e) CRCA-786 classifiers (n=17).

Supplementary Figure S5. PCA and clustering analysis of nCounter assay from the Singapore FF (fresh frozen) cohort. a. PCA analysis of NanoCRCA data for this cohort showing the distribution of samples run in multiple batches (represented as cartridges; n=145). **b.** Heatmap of all 50 (48 CRCA + 2 other) genes measured on the nCounter platform for all samples (n=145). Top bars show the subtypes assigned by NanoCRCA assay and the batch in which each sample was processed. Colours for batch/cartridge are as as in the legend. **c.** PCA analysis of RNA-Seq TPM values for this cohort (n=17). **d.** Heatmap of all 50 (48 CRCA + 2 other) genes measured on the nCounter platform for the 13 samples with matched

RNA-Seq data (n=13). Top bars show the subtypes assigned by the four classifiers NanoCRCA, CRCA-38, CRCA-786 and CMS classifications, and the batch in which each sample was processed. Colours for batch/cartridge are provided. **e.** Bar plot for comparisons between NanoCRCA and other classifications (CRCA-38, CRCA-786 and CMS) showing percent concordance to NanoCRCA classification of samples (n=13).

Supplementary Figure S6. PCA and clustering analysis of nCounter assay from the OriGene cohort. a. PCA analysis of NanoCRCA data for this cohort showing the distribution of samples run in two batches (represented as cartridge; n=17). **b.** Heatmap of all 50 (48 CRCA + 2 other) genes measured on the nCounter platform (n=17). Top bars show the subtypes assigned by the four classifiers - NanoCRCA, CRCA-38, CRCA-786 and CMS classifications, and the batch in which each sample was processed. Colours for batch/cartridge are provided. **c.** PCA analysis of microarray data for the OriGene cohort colored by samples (n=17). **d.** Pie chart showing the proportion of different subtypes (including undetermined samples) from NanoCRCA classification of samples (n=17).

Supplementary Figure S7. PCA and clustering analysis of nCounter assay from the INCLIVA-Valencia cohort. a. PCA analysis of NanoCRCA data for the fresh frozen samples from this cohort showing the distribution of samples run in multiple batches (represented as cartridges; 58 samples with tumor cellularity >10%). **b.** Heatmap of all 50 (48 CRCA + 2 other) genes measured on the nCounter platform in 24 fresh frozen samples having cellularity ≥ 70% in both fresh frozen and FFPE tissues. Top bars show the subtypes assigned by NanoCRCA assay and the batch of the samples (represented as cartridges). Colours for batch/cartridge are provided. **c.** PCA analysis of NanoCRCA data for the FFPE data for this cohort showing the distribution of samples run in multiple batches (represented as cartridges; 58 samples with tumor cellularity >10%). **d.** Heatmap of all 50 (48 CRCA + 2 other) genes measured on the nCounter platform in 24 FFPE samples having cellularity ≥ 70% in both fresh frozen and FFPE tissues. Top bars show the subtypes assigned by NanoCRCA assay and the batch of the samples (represented as cartridges). Colours for batch/cartridge are provided. **e.** Heatmaps showing gene expression of the 38-gene NanoCRCA panel as measured in matched FFPE and fresh frozen tissues with tumour cellularity ≥70% (n=24). Top bars show the subtype as assigned by NanoCRCA in fresh frozen and FFPE tissues. The right-hand vertical bars indicate the subtype association of each gene.

Supplementary Figure S8. PCA and clustering analysis of nCounter assay from the Singapore FFPE cohort. a. PCA analysis of NanoCRCA data for this cohort showing the distribution of samples run in multiple batches (represented as cartridges; n=106). **b.** Heatmap of all 50 (48 CRCA + 2 other) genes measured on the nCounter platform. Top bars show the subtypes assigned by NanoCRCA assay and the batch of the samples (represented as cartridges; n=106). Colours for batch/cartridge are provided in a).

Supplementary Table Legends

Supplementary Table S1. Platform, classifier and cohort information, and list of gene sets used for microarray/RNA-Seq or nCounter assays. a) Overview of relative platform costs and turnaround times. **b)** Overview of expression profile classifiers: derivation, platform, publication in which it was first introduced, and relationships of the classifiers to each other. **c)** Overview of patient cohorts: sample numbers and types, platforms, public data and publications, and patient characteristics. All samples are from surgical specimens collected prior to any treatment. **d)** Original CRCA-786 genes, the 50 genes selected for the custom nCounter panel along with the final 38 genes used for subtype assignment by NanoCRCA, alongside subtype and marker annotation.

Supplementary Table S2. Normalised fresh frozen nCounter data and housekeeping genes for those samples used for assessing protocols and assay reproducibility – fresh frozen samples from Montpellier and OriGene cohorts. a) Normalised log_2 data of samples profiled using standard protocol and **b)** modified protocol (both normalised to HKs: *RPL13a*, *PPIA*, *ZNF384*). **c)** Selected housekeeping genes for normalisation of samples profiled for the reproducibility of the modified protocol assay and **d)** normalised log_2 data of these samples.

Supplementary Table S3. Contingency tables for subtype concordance between protocols in fresh frozen and FFPE samples. Concordance metrics, including percentage subtype concordance and Fisher's exact text results, for subtyping using the standard and modified protocols.

Supplementary Table S4. Normalised FFPE nCounter data and housekeeping genes for those samples used for assessing protocols and assay reproducibility – FFPE samples from RETRO-C cohort. a) Normalised log_2 data 12 of RETRO-C samples profiled using standard protocol and **b)** modified protocol (both normalised to HKs: *RPL13a*, *PPIA*, *ZNF384*). **c)** Selected housekeeping genes for normalisation of samples profiled for the reproducibility of the modified protocol assay and **d)** normalised log_2 data of these samples.

Supplementary Table S5. Training data and misclassification errors for selection of the 38 genes. a) Normalised, log_2 and median centred data for 195 samples used to select the 38 genes and train PAM centroids. **b)** Gene selection using 12 different gene selection/class prediction methods and the corresponding misclassification error rate.

Supplementary Table S6. Final subtype assignments. CRCA-786, CRCA-38, NanoCRCA and CMS subtypes for all samples in all subtyped cohorts.

Supplementary Table S7. Normalised nCounter PanCancer Progression Panel data and housekeeping genes used for normalisation. a) Selected housekeeping genes and **b)** Normalised log_2 data for samples profiled using the PanCancer Progression Panel.

Supplementary Table S8. Contingency tables for subtype concordance between classifiers in fresh frozen samples. a) Concordance metrics, including percentage subtype concordance and Fisher's exact text results, for subtyping using NanoCRCA, CRCA-38, CRCA-786 and CMS classifiers in the pooled Montpellier, Singapore FF, and OriGene cohorts. **b)** Concordance metrics, including percentage subtype concordance and chi-squared test results, for subtyping using CRCA-786 and CMS classifiers in the original Colorectal Cancer Subtyping Consortium (CRCSC) CMS cohort. **c-d)** As **a)** for separate analysis of the **c)** Montpellier, **d)** Singapore FF, and **e)** OriGene cohorts.

Supplementary Table S9. Housekeeping genes and normalised nCounter data for the cohorts. **a)** Selected housekeeping genes and **b)** normalised log_2 data for the Montpellier cohort. **c)** Selected housekeeping and **d)** normalised log_2 data for the Singapore FF cohort. **e)** Selected housekeeping genes and **f)** normalised log_2 data for the OriGene cohort. **g)** Selected housekeeping genes and **h)** normalised log_2 data for the INCLIVA-Valencia FF cohort. **i)** Selected housekeeping genes and **j)** normalised log_2 data for the INCLIVA-Valencia FFPE cohort. **k)** Selected housekeeping genes and **l)** normalised log_2 data for the Singapore FFPE cohort.

References

1 Andrews, S. FastQC: A Quality Control tool for High Throughput Sequence Data. http://www.bioinformatics.babraham.ac.uk/projects/fastqc/ (2016).

2 Li, B. & Dewey, C. N. RSEM: accurate transcript quantification from RNA-Seq data with or without a reference genome. *BMC Bioinformatics* **12**, 1-16, doi:10.1186/1471-2105-12-323 (2011).

3 Li, H. *et al.* The Sequence Alignment/Map format and SAMtools. *Bioinformatics* **25**, 2078-2079, doi:10.1093/bioinformatics/btp352 (2009).

4 Broad Institute. Picard. https://broadinstitute.github.io/picard/ (2015).

5 Guinney, J. *et al.* The consensus molecular subtypes of colorectal cancer. *Nature Medicine* **21**, 1350-1356, doi:10.1038/nm.3967 (2015).

6 Newcombe, R. G. Two-sided confidence intervals for the single proportion: comparison of seven methods. *Stat Med* **17**, 857-872 (1998).

7 Johnson, W. E., Li, C. & Rabinovic, A. Adjusting batch effects in microarray expression data using empirical Bayes methods. *Biostatistics* **8**, 118-127 (2007).

8 Meyer, D., Dimitriadou, E., Hornik, K., Weingessel, A. & Leisch, F. e1071: Misc Functions of the Department of Statistics, Probability Theory Group (Formerly: E1071), TU Wien. https://cran.r-project.org/package=e1071 (2017).

9 Liaw, A. & Wiener, M. Classification and regression by randomForest. *R news* **2**, 18-22 (2002).

10 Dudoit, S., Yang, Y. & Bolstad, B. sma: Statistical microarray analysis. https://cran.r-project.org/package=sma (2011).

11 Tibshirani, R., Hastie, T., Narasimhan, B. & Chu, G. Diagnosis of multiple cancer types by shrunken centroids of gene expression. *Proceedings of the National Academy of Sciences* **99**, 6567-6572 (2002).

Figure S1

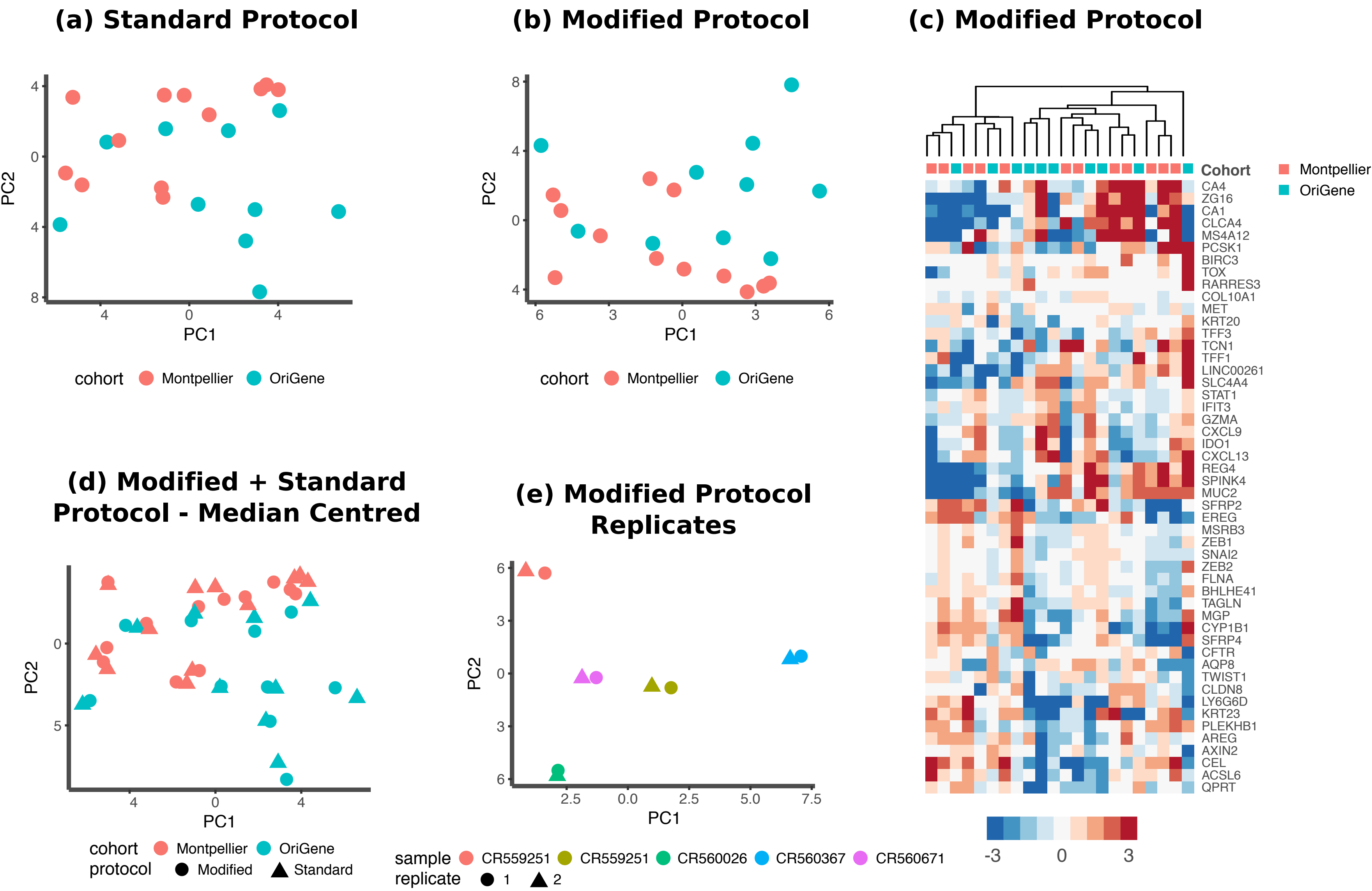

(a) Standard Protocol
(b) Modified Protocol
(c) Modified Protocol
PC2
PC1
cohort
Montpellier
OriGene
Cohort
Montpellier
OriGene
CA4
ZG16
CA1
CLCA4
MS4A12
PCSK1
BIRC3
TOX
RARRES3
COL10A1
MET
KRT20
TFF3
TCN1
TFF1
LINC00261
SLC4A4
STAT1
IFIT3
GZMA
CXCL9
IDO1
CXCL13
REG4
SPINK4
MUC2
SFRP2
EREG
MSRB3
ZEB1
SNAI2
ZEB2
FLNA
BHLHE41
TAGLN
MGP
CYP1B1
SFRP4
CFTR
AQP8
TWIST1
CLDN8
LY6G6D
KRT23
PLEKHB1
AREG
AXIN2
CEL
ACSL6
QPRT
-3
0
3
(d) Modified + Standard Protocol - Median Centred
(e) Modified Protocol Replicates
protocol
Modified
Standard
sample
CR559251
CR559251
CR560026
CR560367
CR560671
replicate
1
2

Figure S2

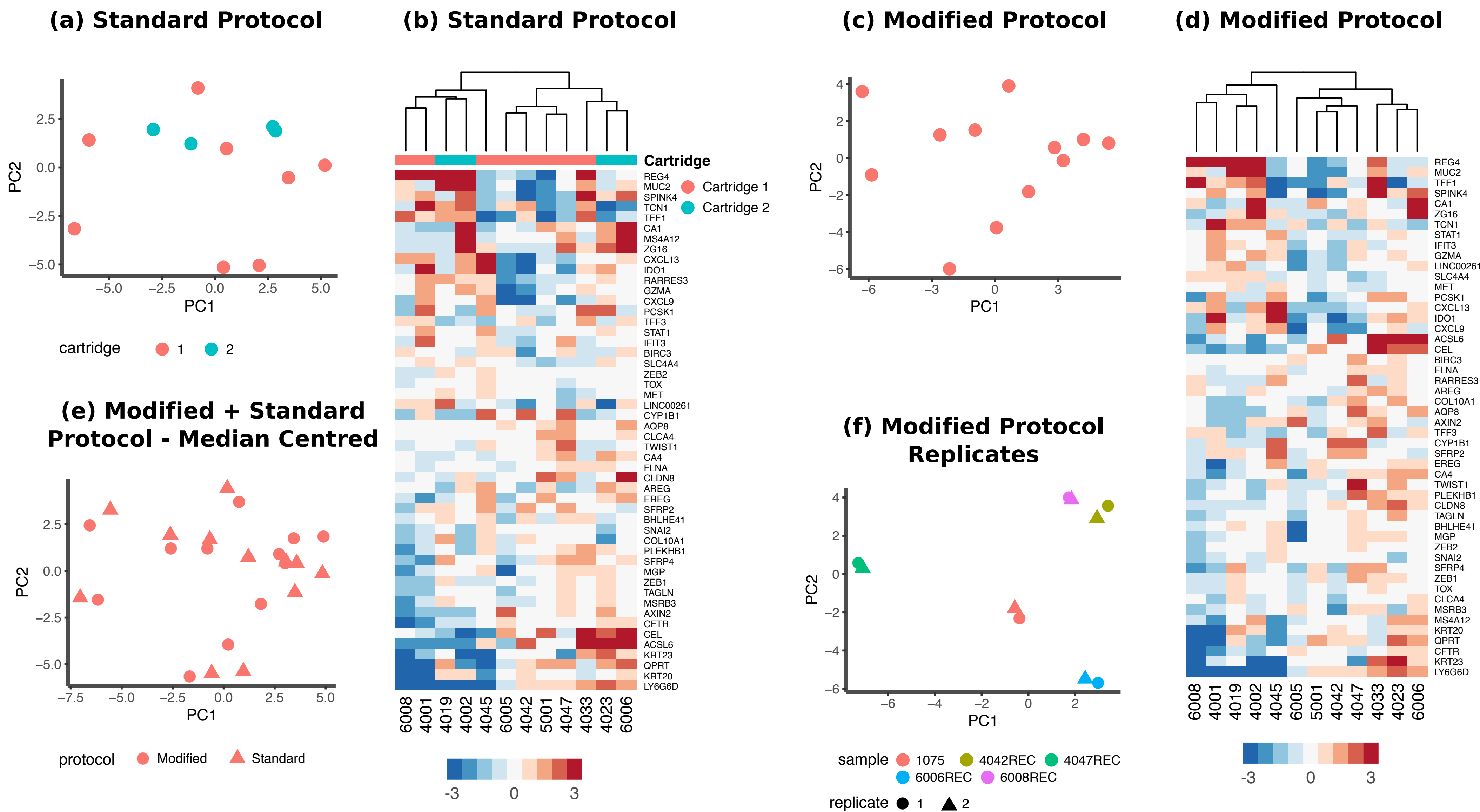

(a) Standard Protocol
PC1
PC2
cartridge 1 2
(b) Standard Protocol
Cartridge
Cartridge 1
Cartridge 2
REG4
MUC2
SPINK4
TCN1
TFF1
CA1
MS4A12
ZG16
CXCL13
IDO1
RARRES3
GZMA
CXCL9
PCSK1
TFF3
STAT1
IFIT3
BIRC3
SLC4A4
ZEB2
TOX
MET
LINC00261
CYP1B1
AQP8
CLCA4
TWIST1
CA4
FLNA
CLDN8
AREG
EREG
SFRP2
BHLHE41
SNAI2
COL10A1
PLEKHB1
SFRP4
MGP
ZEB1
TAGLN
MSRB3
AXIN2
CFTR
CEL
ACSL6
KRT23
QPRT
KRT20
LY6G6D
6008 4001 4019 4002 4045 6005 4042 5001 4047 4033 4023 6006
-3 0 3
(c) Modified Protocol
PC1
PC2
(d) Modified Protocol
REG4
MUC2
TFF1
SPINK4
CA1
ZG16
TCN1
STAT1
IFIT3
GZMA
LINC00261
SLC4A4
MET
PCSK1
CXCL13
IDO1
CXCL9
ACSL6
CEL
BIRC3
FLNA
RARRES3
AREG
COL10A1
AQP8
AXIN2
TFF3
CYP1B1
SFRP2
EREG
CA4
TWIST1
PLEKHB1
CLDN8
TAGLN
BHLHE41
MGP
ZEB2
SNAI2
SFRP4
ZEB1
TOX
CLCA4
MSRB3
MS4A12
KRT20
QPRT
CFTR
KRT23
LY6G6D
6008 4001 4019 4002 4045 6005 5001 4042 4047 4033 4023 6006
-3 0 3
(e) Modified + Standard Protocol - Median Centred
PC1
PC2
protocol Modified Standard
(f) Modified Protocol Replicates
PC1
PC2
sample 1075 4042REC 4047REC 6006REC 6008REC
replicate 1 2

Figure S3

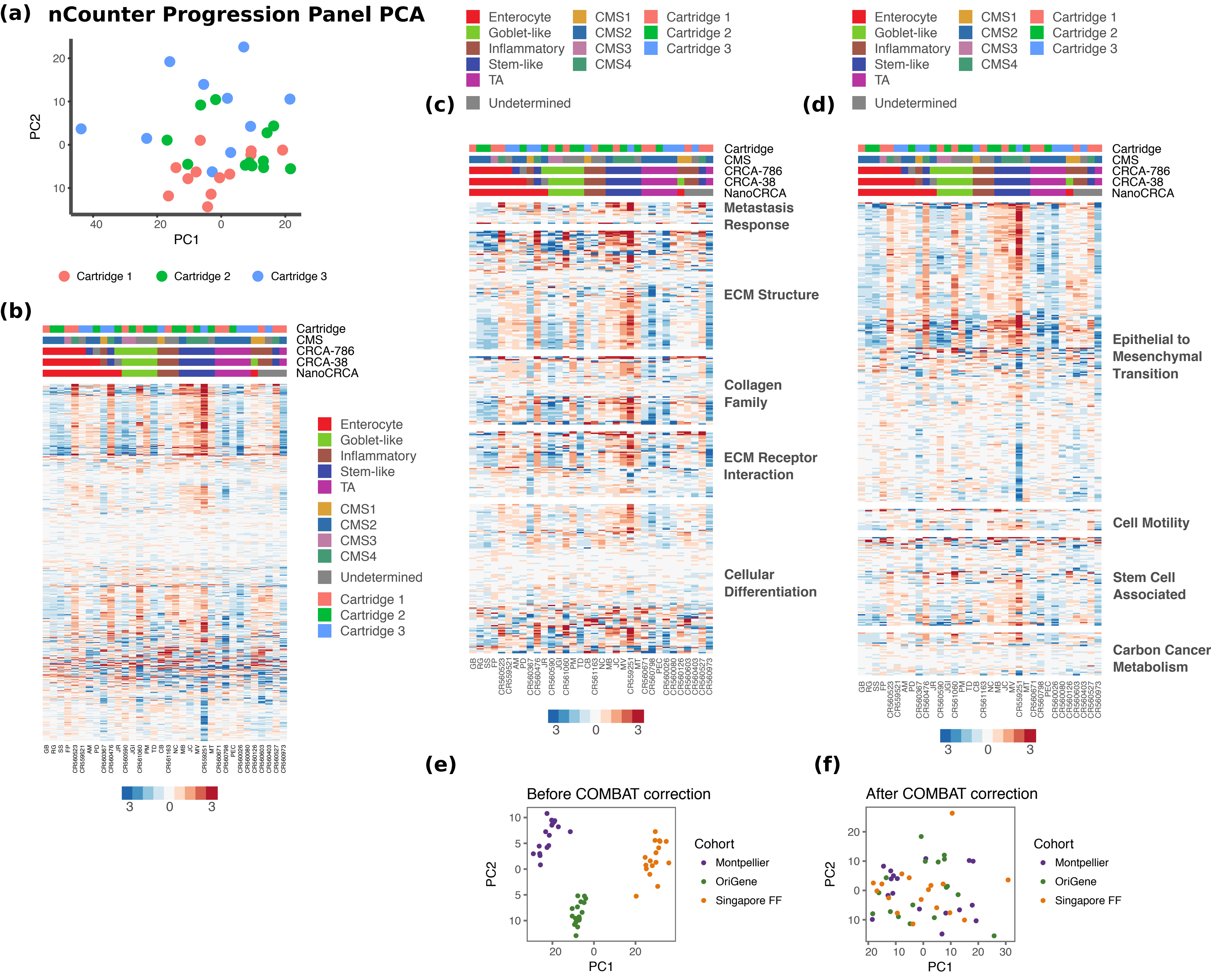
(a)
nCounter Progression Panel PCA
PC2
PC1
Cartridge 1
Cartridge 2
Cartridge 3
(b)
Cartridge
CMS
CRCA-786
CRCA-38
NanoCRCA
Enterocyte
Goblet-like
Inflammatory
Stem-like
TA
CMS1
CMS2
CMS3
CMS4
Undetermined
(c)
Metastasis Response
ECM Structure
Collagen Family
ECM Receptor Interaction
Cellular Differentiation
(d)
Epithelial to Mesenchymal Transition
Cell Motility
Stem Cell Associated
Carbon Cancer Metabolism
(e)
Before COMBAT correction
Cohort
Montpellier
OriGene
Singapore FF
(f)
After COMBAT correction

Figure S4

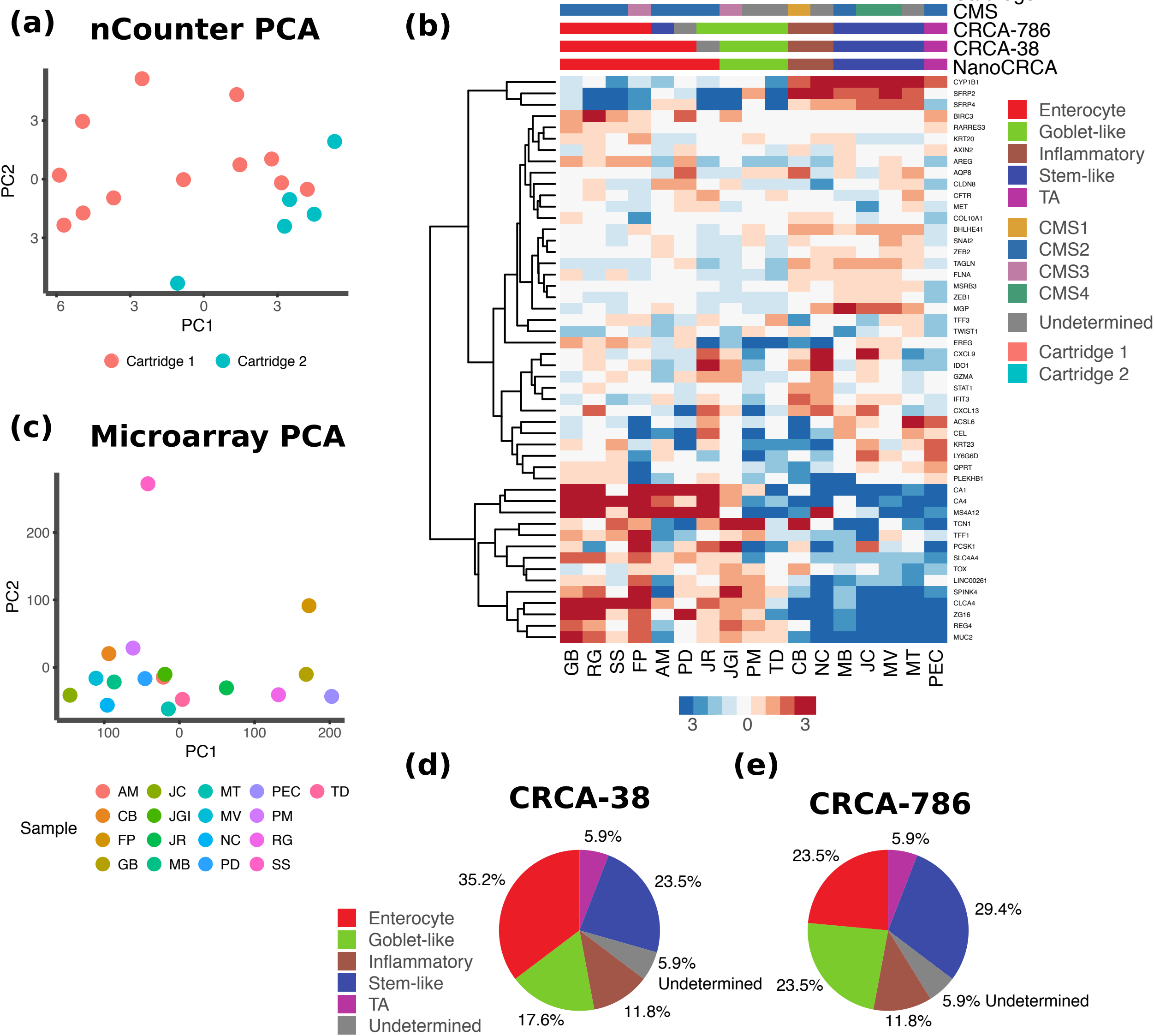
(a)
nCounter PCA
PC2
PC1
Cartridge 1
Cartridge 2
(b)
Cartridge
CMS
CRCA-786
CRCA-38
NanoCRCA
CYP1B1
SFRP2
SFRP4
BIRC3
RARRES3
KRT20
AXIN2
AREG
AQP8
CLDN8
CFTR
MET
COL10A1
BHLHE41
SNAI2
ZEB2
TAGLN
FLNA
MSRB3
ZEB1
MGP
TFF3
TWIST1
EREG
CXCL9
IDO1
GZMA
STAT1
IFIT3
CXCL13
ACSL6
CEL
KRT23
LY6G6D
QPRT
PLEKHB1
CA1
CA4
MS4A12
TCN1
TFF1
PCSK1
SLC4A4
TOX
LINC00261
SPINK4
CLCA4
ZG16
REG4
MUC2
GB RG SS FP AM PD JR JGI PM TD CB NC MB JC MV MT PEC
Enterocyte
Goblet-like
Inflammatory
Stem-like
TA
CMS1
CMS2
CMS3
CMS4
Undetermined
Cartridge 1
Cartridge 2
(c)
Microarray PCA
PC2
PC1
Sample
AM JC MT PEC TD
CB JGI MV PM
FP JR NC RG
GB MB PD SS
(d)
CRCA-38
35.2%
5.9%
23.5%
5.9%
Undetermined
11.8%
17.6%
Enterocyte
Goblet-like
Inflammatory
Stem-like
TA
Undetermined
(e)
CRCA-786
23.5%
5.9%
29.4%
5.9% Undetermined
11.8%
23.5%

Figure S5

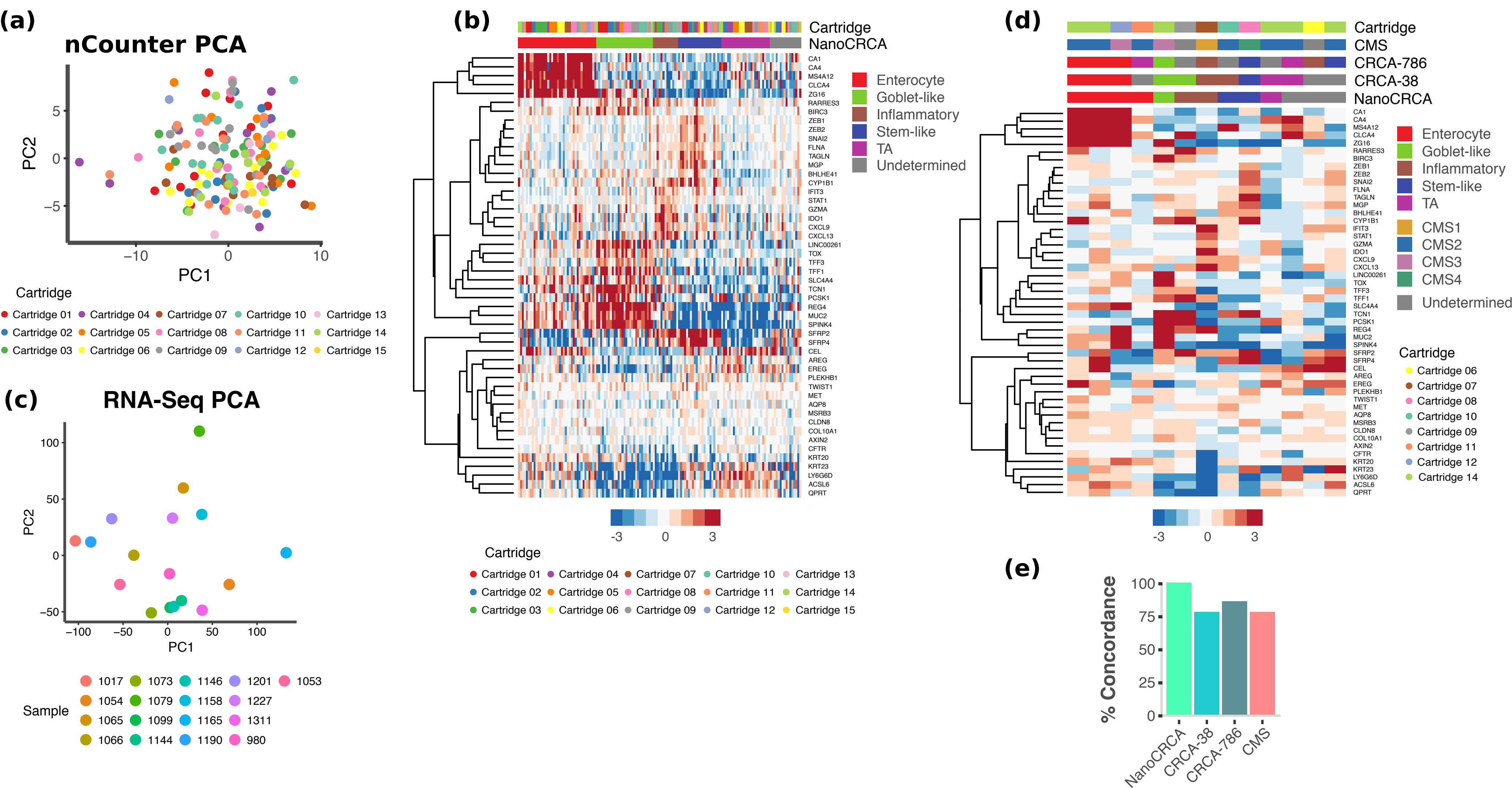

(a)
nCounter PCA
PC2
PC1
Cartridge
Cartridge 01
Cartridge 02
Cartridge 03
Cartridge 04
Cartridge 05
Cartridge 06
Cartridge 07
Cartridge 08
Cartridge 09
Cartridge 10
Cartridge 11
Cartridge 12
Cartridge 13
Cartridge 14
Cartridge 15
(b)
Cartridge
NanoCRCA
Enterocyte
Goblet-like
Inflammatory
Stem-like
TA
Undetermined
CA1
CA4
MS4A12
CLCA4
ZG16
RARRES3
BIRC3
ZEB1
ZEB2
SNAI2
FLNA
TAGLN
MGP
BHLHE41
CYP1B1
IFIT3
STAT1
GZMA
IDO1
CXCL9
CXCL13
LINC00261
TOX
TFF3
TFF1
SLC4A4
TCN1
PCSK1
REG4
MUC2
SPINK4
SFRP2
SFRP4
CEL
AREG
EREG
PLEKHB1
TWIST1
MET
AQP8
MSRB3
CLDN8
COL10A1
AXIN2
CFTR
KRT20
KRT23
LY6G6D
ACSL6
QPRT
(c)
RNA-Seq PCA
Sample
1017
1054
1065
1066
1073
1079
1099
1144
1146
1158
1165
1190
1201
1227
1311
980
1053
(d)
Cartridge
CMS
CRCA-786
CRCA-38
NanoCRCA
CMS1
CMS2
CMS3
CMS4
Undetermined
Cartridge 06
Cartridge 07
Cartridge 08
Cartridge 10
Cartridge 09
Cartridge 11
Cartridge 12
Cartridge 14
(e)
% Concordance
NanoCRCA
CRCA-38
CRCA-786
CMS

Figure S6

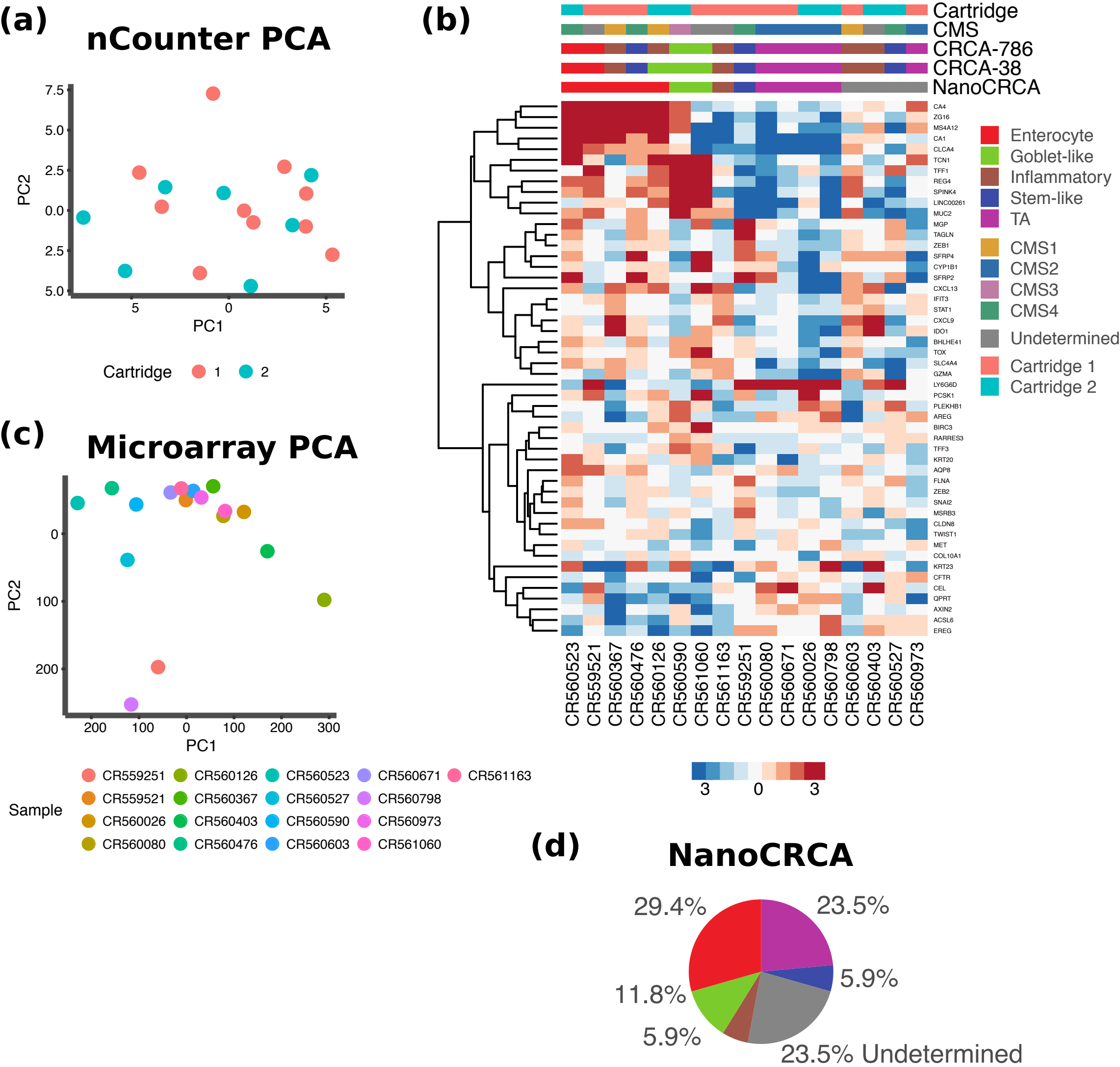

(a)
nCounter PCA
7.5
5.0
2.5
0.0
2.5
5.0
PC2
5
0
5
PC1
Cartridge
1
2
(b)
Cartridge
CMS
CRCA-786
CRCA-38
NanoCRCA
CA4
ZG16
MS4A12
CA1
CLCA4
TCN1
TFF1
REG4
SPINK4
LINC00261
MUC2
MGP
TAGLN
ZEB1
SFRP4
CYP1B1
SFRP2
CXCL13
IFIT3
STAT1
CXCL9
IDO1
BHLHE41
TOX
SLC4A4
GZMA
LY6G6D
PCSK1
PLEKHB1
AREG
BIRC3
RARRES3
TFF3
KRT20
AQP8
FLNA
ZEB2
SNAI2
MSRB3
CLDN8
TWIST1
MET
COL10A1
KRT23
CFTR
CEL
QPRT
AXIN2
ACSL6
EREG
CR560523
CR559521
CR560367
CR560476
CR560126
CR560590
CR561060
CR561163
CR559251
CR560080
CR560671
CR560026
CR560798
CR560603
CR560403
CR560527
CR560973
Enterocyte
Goblet-like
Inflammatory
Stem-like
TA
CMS1
CMS2
CMS3
CMS4
Undetermined
Cartridge 1
Cartridge 2
3
0
3
(c)
Microarray PCA
0
100
200
PC2
200
100
0
100
200
300
PC1
Sample
CR559251
CR559521
CR560026
CR560080
CR560126
CR560367
CR560403
CR560476
CR560523
CR560527
CR560590
CR560603
CR560671
CR560798
CR560973
CR561060
CR561163
(d)
NanoCRCA
29.4%
23.5%
5.9%
11.8%
5.9%
23.5% Undetermined

Figure S7

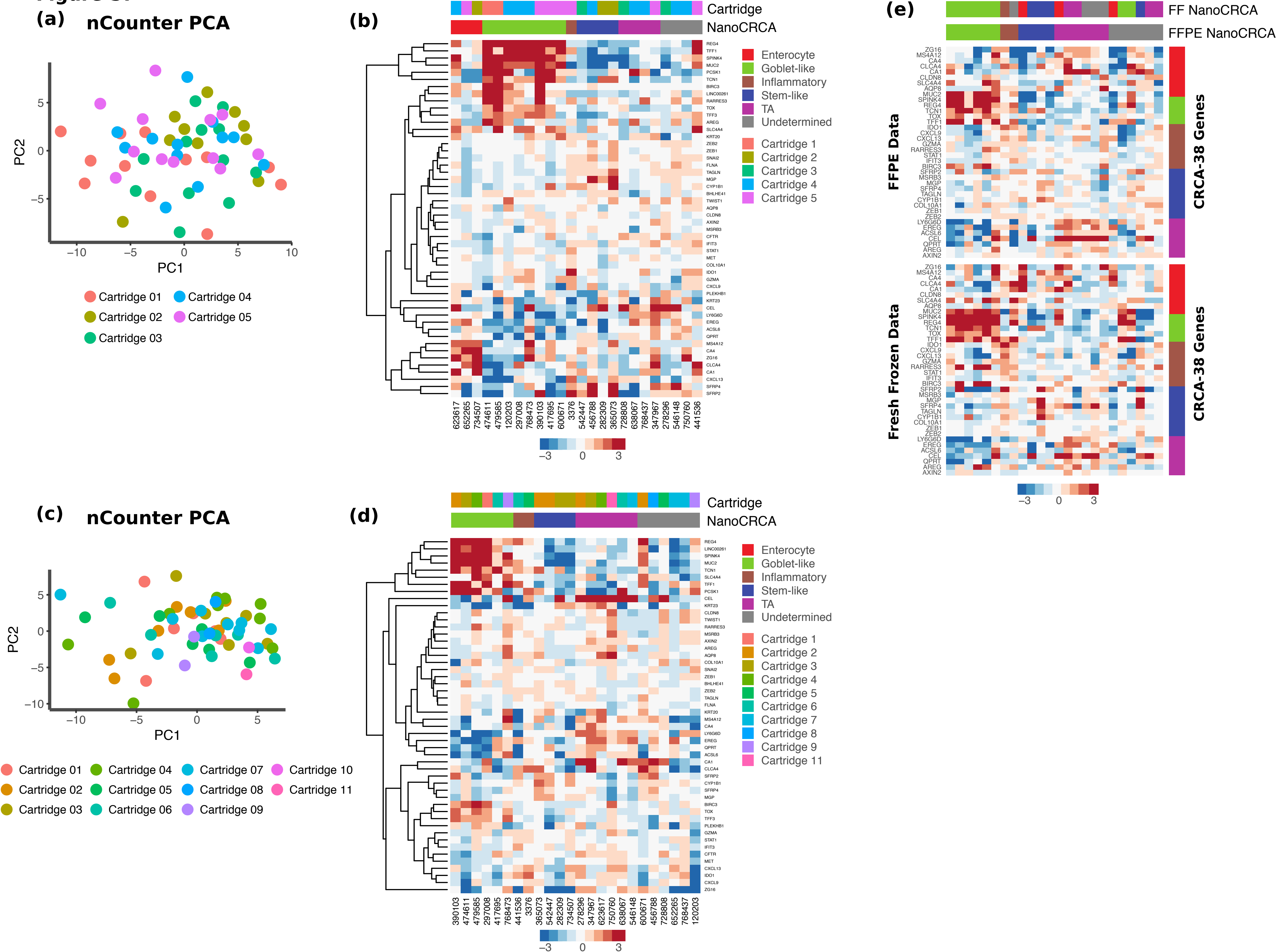
(a)
nCounter PCA
PC2
PC1
Cartridge 01
Cartridge 02
Cartridge 03
Cartridge 04
Cartridge 05
(b)
Cartridge
NanoCRCA
Enterocyte
Goblet-like
Inflammatory
Stem-like
TA
Undetermined
Cartridge 1
Cartridge 2
Cartridge 3
Cartridge 4
Cartridge 5
(c)
nCounter PCA
PC2
PC1
Cartridge 01
Cartridge 02
Cartridge 03
Cartridge 04
Cartridge 05
Cartridge 06
Cartridge 07
Cartridge 08
Cartridge 09
Cartridge 10
Cartridge 11
(d)
Cartridge
NanoCRCA
Enterocyte
Goblet-like
Inflammatory
Stem-like
TA
Undetermined
Cartridge 1
Cartridge 2
Cartridge 3
Cartridge 4
Cartridge 5
Cartridge 6
Cartridge 7
Cartridge 8
Cartridge 9
Cartridge 10
Cartridge 11
(e)
FF NanoCRCA
FFPE NanoCRCA
FFPE Data
CRCA-38 Genes
Fresh Frozen Data
CRCA-38 Genes

Figure S8

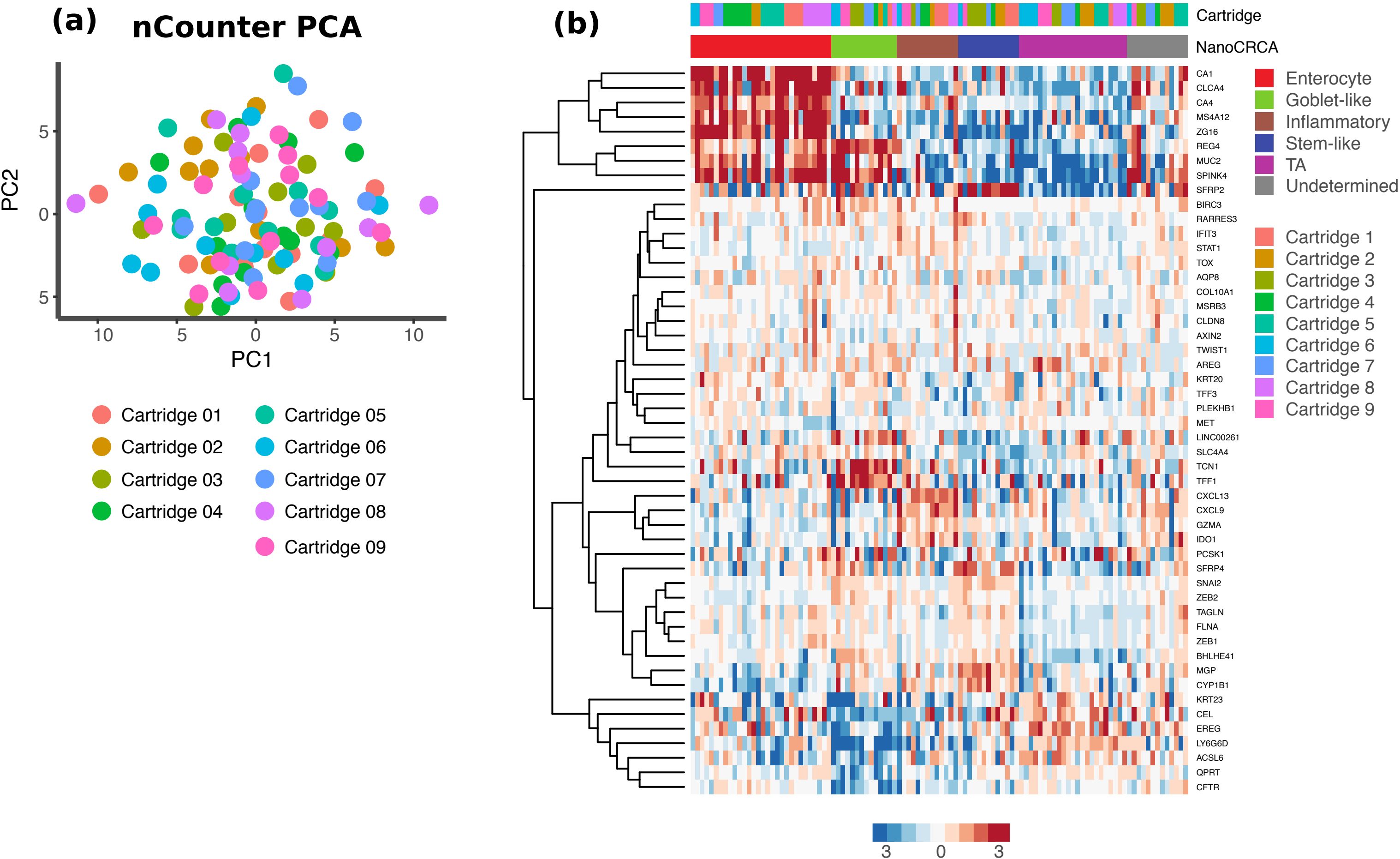

(a)
nCounter PCA
PC2
PC1
5
0
5
10
5
0
5
10
Cartridge 01
Cartridge 02
Cartridge 03
Cartridge 04
Cartridge 05
Cartridge 06
Cartridge 07
Cartridge 08
Cartridge 09
(b)
Cartridge
NanoCRCA
CA1
CLCA4
CA4
MS4A12
ZG16
REG4
MUC2
SPINK4
SFRP2
BIRC3
RARRES3
IFIT3
STAT1
TOX
AQP8
COL10A1
MSRB3
CLDN8
AXIN2
TWIST1
AREG
KRT20
TFF3
PLEKHB1
MET
LINC00261
SLC4A4
TCN1
TFF1
CXCL13
CXCL9
GZMA
IDO1
PCSK1
SFRP4
SNAI2
ZEB2
TAGLN
FLNA
ZEB1
BHLHE41
MGP
CYP1B1
KRT23
CEL
EREG
LY6G6D
ACSL6
QPRT
CFTR
Enterocyte
Goblet-like
Inflammatory
Stem-like
TA
Undetermined
Cartridge 1
Cartridge 2
Cartridge 3
Cartridge 4
Cartridge 5
Cartridge 6
Cartridge 7
Cartridge 8
Cartridge 9
3
0
3
